# Interpretation of correlated neural variability from models of feed-forward and recurrent circuits

**DOI:** 10.1371/journal.pcbi.1005979

**Published:** 2018-02-06

**Authors:** Volker Pernice, Rava Azeredo da Silveira

**Affiliations:** 1 Department of Physics, Ecole Normale Supérieure, Paris, France; 2 Laboratoire de Physique Statistique, Ecole Normale Supérieure, PSL Research University; Université Paris Diderot Sorbonne Paris-Cité, Sorbonne Universités UPMC Univ Paris 06; CNRS, Paris, France; 3 Princeton Neuroscience Institute, Princeton University, Princeton, New Jersey, United States of America; University of Pittsburgh, UNITED STATES

## Abstract

Neural populations respond to the repeated presentations of a sensory stimulus with correlated variability. These correlations have been studied in detail, with respect to their mechanistic origin, as well as their influence on stimulus discrimination and on the performance of population codes. A number of theoretical studies have endeavored to link network architecture to the nature of the correlations in neural activity. Here, we contribute to this effort: in models of circuits of stochastic neurons, we elucidate the implications of various network architectures—recurrent connections, shared feed-forward projections, and shared gain fluctuations—on the stimulus dependence in correlations. Specifically, we derive mathematical relations that specify the dependence of population-averaged covariances on firing rates, for different network architectures. In turn, these relations can be used to analyze data on population activity. We examine recordings from neural populations in mouse auditory cortex. We find that a recurrent network model with random effective connections captures the observed statistics. Furthermore, using our circuit model, we investigate the relation between network parameters, correlations, and how well different stimuli can be discriminated from one another based on the population activity. As such, our approach allows us to relate properties of the neural circuit to information processing.

## Author summary

The response of neurons to a stimulus is variable across trials. A natural solution for reliable coding in the face of noise is the averaging across a neural population. The nature of this averaging depends on the structure of noise correlations in the neural population. In turn, the correlation structure depends on the way noise and correlations are generated in neural circuits. It is in general difficult to identify the origin of correlations from the observed population activity alone. In this article, we explore different theoretical scenarios of the way in which correlations can be generated, and we relate these to the architecture of feed-forward and recurrent neural circuits. Analyzing population recordings of the activity in mouse auditory cortex in response to sound stimuli, we find that population statistics are consistent with those generated in a recurrent network model. Using this model, we can then quantify the effects of network properties on average population responses, noise correlations, and the representation of sensory information.

## Introduction

In the search for clues about the function of neural circuits, it has become customary to rely upon recordings of the activity of large populations of neurons. These measurements exhibit the concerted responses of neurons in different conditions, such as presentations of different stimuli. With the stimulus-dependent, high-dimensional statistics of neural responses in hand, one can ask two questions: How are these statistics generated in the neural population? What purpose, if any, do they serve? While the first question is mechanistic and the second is functional, the two are intimately linked. We address these questions by relating observed population activity to the output of different circuit models. In particular, we examine the correlated response variability that arises from diverse circuit architecture, and, in turn, its effect on stimulus representation.

Along the mechanistic line of research, a number of network models have been proposed to explain the relation between anatomical and physiological parameters, on the one hand, and the noise correlations, on the other hand. The way in which correlations emerge, and may be suppressed, in recurrent networks was analyzed in detail with simplified biophysical models [1–4], as well as more abstracted models using binary neurons [5] or Poisson neurons [6]. As an alternative to recurrent connections, shared external input (e.g., from top-down afferents) or shared gain fluctuations can also be at the origin of correlations in neural populations. This and similar mechanisms were exploited in recent studies [7, 8], in particular to account for observations in both visual cortex [9–12] and retina [13, 14].

In modeling stochastic neural activity, models of Poisson neurons or generalizations thereof have proved eminently useful. These have been employed to investigate experimentally measured statistics in the context of both feed-forward [15] and recurrent networks [16], as well as in the context of models with shared gain fluctuations [9, 13]. Poisson models can capture a wide range of statistics; their parameters can be viewed as an abstraction from the biophysical properties of neurons.

An important motivation to understand the origin of the statistics of neural activity is the latter’s connection with the representation of sensory information in neural populations (for a recent review, see Ref. [17]). A much studied example is that of the influence of recurrent connections on the properties of the mean response of neurons, or tuning curves [18–20]. Beyond the study of first-order statistics, a great deal of work has been devoted to the investigation of the relation between correlated variability in population response and the accuracy of stimulus representation. Several classic studies have pointed to the fact that noise correlations can be harmful to information coding [21–23], while a number of more recent studies indicate that noise correlation can also have a beneficial impact on coding, depending on its fine structure [23–26]. For example, the effect of noise correlation can depend appreciable on its stimulus dependence [13, 14] or on the physiological heterogeneity present in the population [27–29]. The way in which noise correlations arise from circuit properties has been examined in simulated networks [18] and analyzed theoretically in the framework of Poisson neurons [30, 31]. Recent work has also investigated the limits on the representation of information imposed by properties of neural circuits, via the structure of correlations these induce [32, 33].

Here, we aim at relating possible mechanistic origins of correlation to the statistics of neural population activity, on the one hand, and at relating circuit properties to the representation of information, on the other hand. Rather than starting from a specific circuit model or relying on simulations of a certain network architecture, we want to be able to infer circuit properties of fairly general models for large and interconnected populations from observed activity. We achieve these aims by establishing mathematical expressions that relate circuit properties to population activity statistics; in particular, to variances and covariances in the population. These expressions connect the various parts of our investigation. We obtain these expressions in the framework of the Hawkes model [34], made up of coupled Poisson neurons.

We examine three types of circuit architectures, namely, recurrent networks, feed-forward networks, and networks with shared gain fluctuations. Instead of focusing on specific realizations of these networks, however, we consider ensembles of such networks, defined through distributions of random connections. This allows us to identify generic signatures of circuit architectures in the correlation structures they induce, which can be used to distinguish them based on recorded activity. We then analyze experimental data, namely, populations recordings in mouse auditory cortex [35], in the light of our model results, and we show that the structure of noise correlations in auditory cortex is in agreement with predictions from a recurrent network. Finally, we turn to the relation between circuit architecture and information coding. Using the derived mathematical expressions, we analyze the effect of network parameters on the representation of information, in the case of different circuit architectures.

## Materials and methods

### Experimental data set

The data set was first reported on in Ref. [35] which discussed the structure of the average population responses as a function of stimulus. The data consists of the activity of neural populations (46-99 neurons) recorded with calcium imaging in the auditory cortex of mice. Animals were isoflurane anesthetized (1%). Signals were obtained from neurons labeled with the synthetic calcium indicator OGB1. Fluorescence was measured at 30 Hz sampling rate, and firing rates were inferred from temporal deconvolution of the fluorescence signal. Up to 6 neural populations in each of 14 animals were recorded. The data points we use are the average firing rates over a window of 250 ms after presentation of each of 65 different sound stimuli, each for 15 trials. Sound stimuli consisted in 50-70 ms presentations of pure frequency tones or complex broadband sounds generated from animal calls or musical pieces. Onset and offset were smoothed, and the stimuli were played at different volumes. Thus, the data points represent population responses evoked by a short sound presentation, time-averaged over a 250 ms window. Responses were measured relative to spontaneous activity; hence, negative responses occurred if the stimulus-evoked firing rates were smaller than the spontaneous ones.

### Measures of response variability

While the precise timing of spikes can convey a large amount of information [36], in many studies this temporal dimension is neglected, and the representation of stimuli is considered in terms of spike counts in given time windows. This approach is legitimate in cases in which sensory coding occurs on a relatively slow time scale; here, we also assume this form of spike count coding. We denote the vector of population activity in trial *T* of stimulus *s* is by *r*(*s*, *T*), and the average response across trials for this stimulus by *r*(*s*) ≡ 〈*r*(*s*, *T*)〉_*T*_. If not explicitly stated otherwise, here, and throughout, we consider time-averaged neural responses, both when referring to experimental data and in the theoretical analysis; i.e., the vector *r*(*s*, *T*) represents the neural activity averaged across a chosen time window. To measure covariability between neurons *i* and *j* across trials, in response to a given stimulus, we use the noise covariances, defined as

$$\begin{array}{c} C_{ij}\left(s\right)=\text{cov}{\left(r_{i}\left(s,T\right),r_{j}\left(s,T\right)\right)}_{T}={\left\langle r_{i}\left(s,T\right)r_{j}\left(s,T\right)\right\rangle }_{T}-{\left\langle r_{i}\left(s,T\right)\right\rangle }_{T}{\left\langle r_{j}\left(s,t\right)\right\rangle }_{T}. \end{array}$$ (1)

A measure of the strength of pairwise noise correlations across stimuli is the average correlation coefficient, defined as

$$c_{ij}^{N}={\langle \frac{C_{ij}\left(s\right)}{\sqrt{C_{ii}\left(s\right)C_{jj}\left(s\right)}}\rangle }_{s},$$ (2)

where 〈.〉_*s*_ denotes the average over all stimuli presented. This quantity is to be contrasted with the signal correlation,

$$\begin{array}{c} c_{ij}^{S}=\frac{\text{cov}{\left(r_{i}\left(s\right),r_{j}\left(s\right)\right)}_{s}}{\sqrt{\text{var}{\left(r_{i}\left(s\right)\right)}_{s}\text{var}{\left(r_{j}\left(s\right)\right)}_{s}}}, \end{array}$$ (3)

which measures the similarity of average responses across stimuli. To quantify the way in which the orientation of the high-dimensional distribution changes across stimuli, we use the variance of the population activity projected along the direction of the (normalized) mean response, $\bar{r}\left(s\right)=r\left(s\right)/\left|r\left(s\right)\right|$, i.e.,

$$\begin{array}{c} \sigma_{\mu }^{2}\left(s\right)=\sum_{ij}C_{ij}\left(s\right){\bar{r}}_{j}\left(s\right){\bar{r}}_{i}\left(s\right). \end{array}$$ (4)

**Fig 1:**
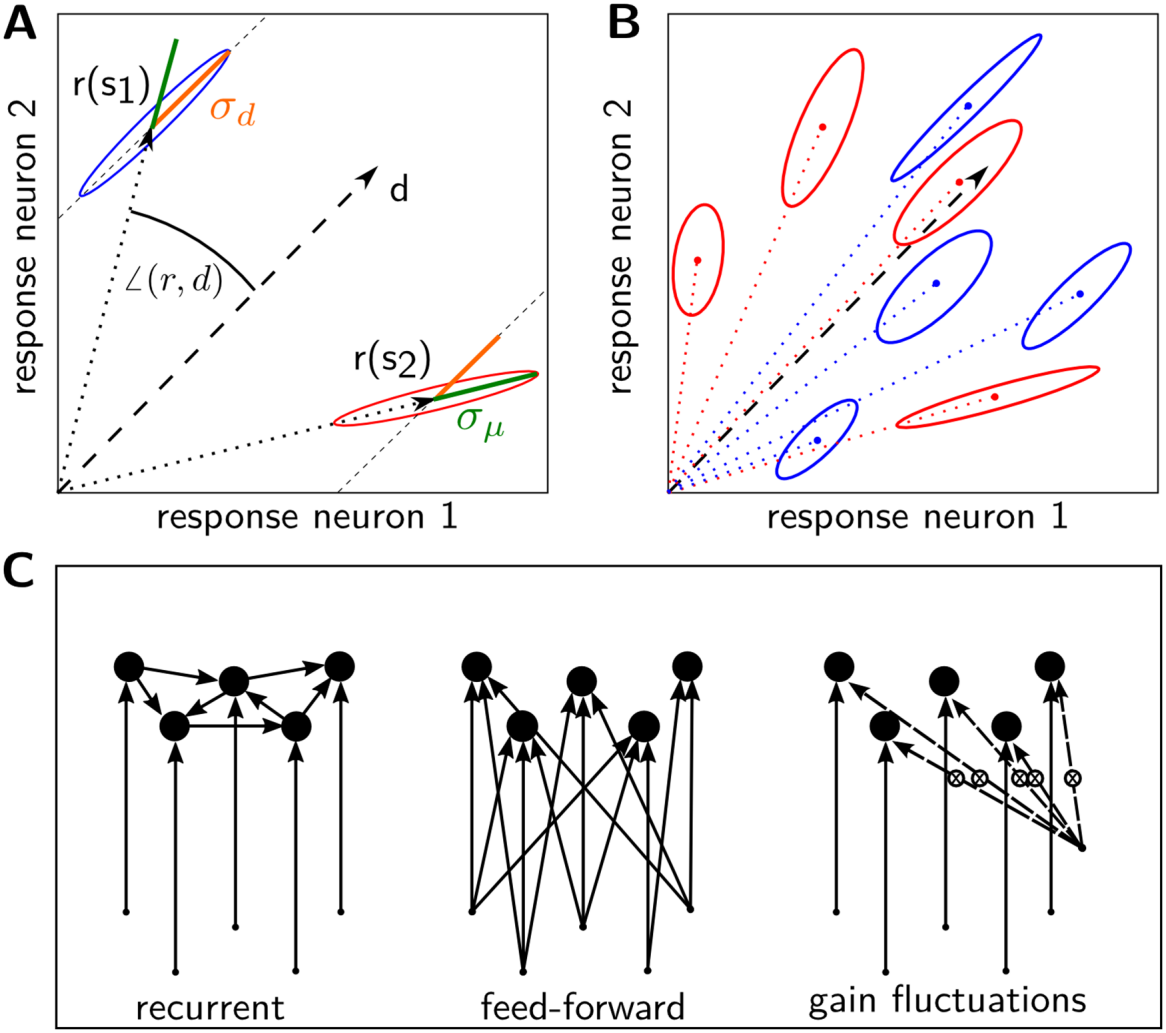
Properties of response distributions and network scenarios. A: Examples of distributions for which the variability is mostly along the diagonal direction (blue ellipse), resulting in a large value of *σ*_*d*_, and for which the variability is mostly along the direction of the average response (red ellipse), resulting in a large value of *σ*_*μ*_. B: Response distributions for two stimulus ensembles. Either *σ*_*μ*_ (red set) or *σ*_*d*_ (blue set) remains large across stimuli. A dependence as in the red set emerges in feed-forward models with common gain fluctuations, while a dependence as in the blue set may arise in densely connected recurrent networks or in feed-forward networks with shared inputs. C: Different network architectures which induce correlated activity. Connections (arrows) to and between neurons (dots) vary in strength. Dashed arrows indicate multiplicative modulations.

This quantity can be compared to the variance projected along the diagonal direction, $\bar{d}={\left(1,\ldots ,1\right)}^{T}/\sqrt{N}$,

$$\begin{array}{c} \sigma_{d}^{2}\left(s\right)=\sum_{ij}C_{ij}\left(s\right){\bar{d}}_{i}{\bar{d}}_{j}, \end{array}$$ (5)

which corresponds to a uniform averaging of the covariances. To compare these quantities across stimuli, $\sigma_{\mu }^{2}$ and $\sigma_{d}^{2}$ are normalized by the sum of the variances, $\sigma_{all}^{2}\left(s\right)=\sum_{i}C_{ii}\left(s\right)$. If, for a given stimulus, all neurons are equally active on average, then $\bar{r}=\bar{d}$ and $\sigma_{d}^{2}=\sigma_{\mu }^{2}$. As we show below, different circuit models predict distinct stimulus dependencies of $\sigma_{\mu }^{2}$ and $\sigma_{d}^{2}$. The discrepancies are most apparent for the stimuli for which the average population response differs strongly from a uniform population response. A deviation from a uniform response can be measured by the angle between mean response and diagonal, or $\cos\left(d,r\right)={\bar{r}}^{T}\cdot \bar{d}$. For a graphical illustration of these measures, see Fig 1A and 1B.

### Recurrent model of Poisson neurons with noisy input

#### Definition of the model and derivation of its statistics

We model an intrinsically noisy process of spike generation in neurons, in which the effect of presynaptic spikes on neural activity is captured through linear modulations of an underlying firing rate. Although the results in this paper refer to time-averaged quantities, in this section we consider time-dependent quantities for definitions and derivations; time dependence is indicated explicitly by the notation $\tilde{\cdot }\left(t\right)$. The spike train of neuron *i* is a realization of an inhomogeneous Poisson process with a time-dependent firing rate, ${\tilde{r}}_{i}\left(t\right)$, calculated as

$$\begin{array}{c} {\tilde{r}}_{i}\left(t\right)={\tilde{r}}_{\text{ext},i}\left(t\right)+\sum_{j}\int_{0}^{\infty }{\tilde{g}}_{ij}\left(\tau \right){\tilde{s}}_{j}\left(t-\tau \right)d\tau , \end{array}$$ (6)

where ${\tilde{r}}_{\text{ext},i}\left(t\right)$ is the external input, ${\tilde{s}}_{j}\left(t\right)=\sum_{t^{\prime }}\delta \left(t-t^{\prime }\right)$ is the recurrent input from presynaptic neuron *j*, and the causal coupling kernel, ${\tilde{g}}_{ij}\left(\tau \right)$, defines the interactions between neurons in the network. The external input is an analog quantity which can be viewed as resulting from a convolution of a linear filter and an incoming spike train from ‘non-local’ presynaptic neurons. We treat it as a noisy, but stationary, signal, i.e., its mean and higher moments do not depend on time. While this model of interacting point processes [34] defines the full temporal dynamics of the system, we are only interested in time-averaged rates or, equivalently, spike counts. Their expected values and (co-)variances across realizations can be obtained if the coupling matrix is known.

Assuming stationarity of input firing rates, we can solve for the average rate vector across trial realizations, *r* = (*r*_1_,…*r*_*N*_), as

$$\begin{array}{c} r={\left(I-G\right)}^{-1}r_{\text{ext}}. \end{array}$$ (7)

Here, I denotes the identity matrix, *G* is the steady-state coupling matrix with elements

$$\begin{array}{c} G_{ij}=\int_{0}^{\infty }{\tilde{g}}_{ij}\left(\tau \right)d\tau , \end{array}$$ (8)

and *r*_ext_ is the average input vector. To describe the correlation in the activity, we consider the spike count in a large time bin of duration Δ, defined as

$$\begin{array}{c} n_{i}\left(\Delta \right)=\int_{t}^{t+\Delta }{\tilde{s}}_{i}\left(t\right)dt. \end{array}$$ (9)

The total input in the time bin is given by

$$\begin{array}{c} r_{\text{ext}}\left(\Delta \right)=\int_{t}^{t+\Delta }{\tilde{r}}_{E}\left(t\right)dt, \end{array}$$ (10)

and is a random variable with normalized variance *V*_ext_ ≡ *var*(*r*_ext_(Δ))/Δ (across choices of different time bins, or, equivalently, over trials). From spectral properties of the covariance [34] and the relation between covariance and count correlations [6, 37], it can be shown that the count covariance normalized by the time bin duration, *C*, with elements

$$\begin{array}{c} C_{ij}\equiv \lim_{\Delta \to \infty }\frac{\text{cov}(n_{i}\left(\Delta \right),n_{j}\left(\Delta \right))}{\Delta }, \end{array}$$ (11)

is given by the matrix

$$\begin{array}{c} C={\left(I-G\right)}^{-1}(D\left[r\right]+D\left[V_{\text{ext}}\right]){\left(I-G^{T}\right)}^{-1}. \end{array}$$ (12)

The term *D*[*r*] denotes a diagonal matrix with elements *D*[*r*]_*ij*_ = *r*_*i*_ *δ*_*ij*_, and similarly *D*[*V*_ext_]_*ij*_ = *δ*_*ij*_ *V*_ext,*i*_. The effect of the recurrent network interactions is reflected in the transfer matrix,

$$\begin{array}{c} B\equiv {\left(I-G\right)}^{-1}. \end{array}$$ (13)

In Eq (12), one component of the covariance arises from the variance of the external input, *V*_ext_. Although the intrinsic noise from the spike-generating Poisson process is dynamical, its effect is that of an additive contribution to the external variance (see also Ref. [38]). The dependence on the rate vector, in Eq (12), results from the Poisson character of the noise, in which the variance equates the mean.

In the experimental data set, firing rates are measured relative to spontaneous activity. To take this into account, an offset, *a*, can be added to the rates in Eq (12). In the remainder, we examine the model through Eqs (7) and (12) for the mean neural activities and their covariances, respectively.

#### Random effective network and stimulus ensemble

The rates and covariances given in Eqs (7) and (12) depend on the recurrent coupling matrix of the network, *G*, only via effective couplings defined by the transfer matrix, $B={\left(I-G\right)}^{-1}$. In numerical calculations, we choose the entries of *G* independently from a normal distribution with positive mean. We note that we assume the same distribution for diagonal and off-diagonal elements of *G*; as long as the size of the matrix *G* is sufficiently large, excluding autapses in *G* does not modify the results appreciably. This choice for the random matrix *G* affords a specific structure to the matrix *B*. The empirical mean and variance of the elements of this matrix will be called 〈*B*〉 and *var*(*B*). In a number of analytical calculations, for the sake of simplicity, we neglect the specific structure of *B* and model it as a random matrix with elements independently drawn from a normal distribution with corresponding mean and variance. The elements of *B* are not direct, but effective connections, because they reflect also the effects of indirect connections in the network, hence the expression “random effective network.” The variability of effective connections can be quantified by the ratio *ρ* = *var*(*B*)/〈*B*〉^2^.

The stimulus ensemble is modeled as a set of random vectors, *r*_ext_(*s*). We assume that the elements of these vectors are independent across neurons and stimuli, and normal, and we call the mean and variance of the corresponding normal distribution 〈*r*_ext_〉 and *var*(*r*_ext_). The relative variability of the inputs is defined as *ρ*_ext_ = *var*(*r*_ext_)/〈*r*_ext_〉^2^. This variability is high if both excitatory and inhibitory inputs are present, and, together with the network parameters, it determines the variability of the average responses across stimuli. In numerical calculations, we set *V*_ext,*i*_(*s*) = |*r*_ext,*i*_(*s*)|, as dictated by a Poisson process. We allow for input *signal* correlations, i.e., the average inputs to a pair of neurons can come with a non-vanishing correlation coefficient, *c*_in_ = 〈*r*_ext,*i*_(*s*)_*i*_, *r*_ext,*j*_(*s*)〉_*s*_.

### Models of correlated activity from shared inputs or gain modulations

Correlations in the activity of neurons can have other origins besides recurrent connections. In parallel with the recurrent network model, we consider two alternative prototypical models in which correlations originate from shared inputs or from shared gain fluctuations, respectively. The three different scenarios are illustrated in Fig 1C. In S1 Appendix we show how, formally, these models can also be cast as special cases of the recurrent model.

#### Feed-forward model with shared input

We consider a simple, two-layer, feed-forward network, in which *N* output neurons receive inputs from *N* independent presynaptic neurons with spike trains ${\tilde{s}}_{j}\left(t\right)$. The contributions of the input neurons to the firing rates of the output neurons are determined by feed-forward connection kernels, ${\tilde{f}}_{ij}\left(\tau \right)$, so that the output firing rates are given by

$$\begin{array}{c} {\tilde{r}}_{i}\left(t\right)=\sum_{j}\int_{0}^{\infty }{\tilde{f}}_{ij}\left(\tau \right){\tilde{s}}_{j}\left(t-\tau \right)d\tau . \end{array}$$ (14)

As we show in S1 Appendix, formally this scenario represents a special case of the framework described in the previous section, so that firing rates and count covariances can be calculated correspondingly. We define the feed-forward coupling matrix *F* by its elements,

$$\begin{array}{c} F_{ij}=\int_{0}^{\infty }{\tilde{f}}_{ij}\left(\tau \right)d\tau . \end{array}$$ (15)

If we denote the time-averaged input rates for stimulus *s* by *r*_ext_(*s*), then the average output firing rates are given by

$$\begin{array}{c} r\left(s\right)=Fr_{\text{ext}}\left(s\right). \end{array}$$ (16)

If the spike trains of the input neurons are Poisson processes, the input variance is equal to the rate, *V*_ext_ = *r*_ext_; we will also allow for more general inputs. The count covariances are then calculated as

$$\begin{array}{c} C=FD\left[V_{\text{ext}}\right]F^{T}+D\left[r\right], \end{array}$$ (17)

where we denote again by *D*[*r*] and *D*[*V*_ext_] the diagonal matrices with diagonal elements given by the vectors *r* and *V*_ext_ respectively. The first term describes covariances resulting from the combined shared inputs. The second term results from the contribution to the count variances of the Poisson spike generation in output neurons, which is independent across neurons.

If rates are observed only up to a stimulus-dependent offset, *a*, Eq (17) has to be replaced by

$$\begin{array}{c} C=FD\left[V_{\text{ext}}\right]F^{T}+D\left[r+a\right]. \end{array}$$ (18)

This scenario can be compared directly to the random effective recurrent network model, if one uses *F* = *B* and for an identical ensemble of stimuli (See Sec. “Random effective network and stimulus ensemble”). Comparing Eqs (18) to (12), we note that the difference between the two models is that the internally generated noise in the feed-forward model, *D*[*r* + *a*], is uncorrelated and contributes only to the variances, while in the recurrent network it is filtered by the network and therefore correlated.

#### Feed-forward model with shared gain fluctuations

In addition to recurrence and shared inputs, shared gain fluctuations have also been proposed as a possible source of correlation in neural populations [7, 9, 13, 39]. In this model, the firing rate of the neural population is given by the product of a constant, stimulus-dependent vector, *r*(*s*), and a fluctuating, scalar signal, *ϵ*(*t*). We set the averaged value of the fluctuations to unity, 〈*ϵ*(*t*)〉_*t*_ = 1, so that the averaged firing rate is *r*. The resulting count covariance matrix is given by

$$\begin{array}{c} C=D\left[r\right]+rr^{T}V_{\text{ext}}, \end{array}$$ (19)

where the scalar variance, *V*_ext_, reflects the strength of the variation of the fluctuating signal. In other words, the firing rate of a Poisson neuron is modulated multiplicatively by a fluctuating ‘gain’ signal. One consequence is that the covariance of a neuron pair is proportional to the product of their firing rates. The general model described in Sec. “Recurrent model of Poisson neurons with noisy input” reduces to this model if, for any given stimulus, the activity of the population results from a single input neuron projecting to the output neurons (see S1 Appendix).

Here, the average responses can be chosen freely. When comparing this scenario to the recurrent and feed-forward scenarios, we use the ensemble of average responses resulting from the corresponding network scenario. If the stimulus dependence of the firing rates is measured relative to an offset *a*, the covariances are given by

$$\begin{array}{c} C=D\left[r+a\right]+\left(r+a\right){\left(r+a\right)}^{T}V_{\text{ext}}. \end{array}$$ (20)

### Stimulus discriminability

In order to evaluate the influence of correlation on neural coding, we examine the discriminability of a set of stimuli from the population activity. In the models, a stimulus, *s*, evokes activity characterized by an average response vector, *r*(*s*), and a covariance matrix, *C*(*s*). We seek a simple measure for evaluating the possibility of attributing a given population response to one of two discrete stimuli, *s*_1_ and *s*_2_, unambiguously. For this, we assume that the high-dimensional distributions of responses are Gaussian, and project the two distributions on a single dimension in which we calculate the signal-to-noise ratio as our measure.

The best projection follows from Fisher’s linear discriminant analysis: the most informative linear combination of neuron responses, denoted by *w* ⋅ *r*, is achieved if the vector *w* points in the most discriminant direction,

$$\begin{array}{c} w={\left(C\left(s_{1}\right)+C\left(s_{2}\right)\right)}^{-1}(r\left(s_{1}\right)-r\left(s_{2}\right)). \end{array}$$ (21)

The mean and variance of the projected distributions onto the normalized direction, $\bar{w}$, are ${\bar{w}}^{T}r\left(s_{i}\right)$ and $\sigma_{s_{i}}^{2}={\bar{w}}^{T}C\left(s_{i}\right)\bar{w}$, for *i* ∈ {1, 2}. We can then define the signal-to-noise ratio, as

$$\begin{array}{c} S=\frac{\left|{\bar{w}}^{T}\cdot (r\left(s_{1}\right)-r\left(s_{2}\right))\right|}{\sigma_{s_{1}}+\sigma_{s_{2}}}. \end{array}$$ (22)

Larger values of *S* correspond to better discriminability.

To quantify the effect of correlation on discriminability, we compare the quantity *S*, defined in Eq (22), with the quantity *S*_shuffled_ obtained from a ‘shuffled data set’, in which responses are shuffled across trials (in experimental data) or off-diagonal covariance elements, *C*_*i* ≠ *j*_(*s*), are set to 0 (in models). According to this procedure, we compare the accuracy of the code in the full (experimental or model) data with that in artificially generated data in which noise correlations are removed but which keeps the average populations responses and single-neuron variances unchanged. A ratio *S*_shuffled_/*S* smaller than unity indicates that correlation is beneficial to discriminability.

We note that *S* is an approximation for a measure based on the optimal linear classifier, for which the threshold separating the two one-dimensional projected distributions has to be calculated numerically. A measure akin to *S* used in similar contexts is the linear Fisher information [23], valid for continuous stimuli. An advantageous property of *S* is its invariance under linear transformations: if all responses, *r*, are fed into another network whose output, *Br*, results from a product with an invertible matrix *B*, *S* does not change. This obtains because the mean responses are transformed into *Br*(*s*), while the covariances are transformed into *BC*(*s*)*B*^*T*^. Intuitively, a simple matrix multiplication is accommodated for by a corresponding change in the most discriminant direction *w* on which we project the population activity.

## Results

We discuss the characteristics of the noise statistics that emerge from neural dynamics in models of recurrent networks, feed-forward networks, and networks with global gain fluctuations. We also look for coarse statistical signatures of each of these three model structures, which can be compared with data. A summary of variables defined in the Methods section is presented in Table 1, and a number of key results are summarized in Table 2 which can serve as a guide for contrasting models. In this perspective, we analyze recordings of activity in auditory cortex, in mouse, in response to a battery of sound stimuli [35]. We then discuss the implications of the neural noise statistics on stimulus encoding. Finally, we discuss a highly simplified model of network dynamics, paired down to include only a few parameters, in order to develop an intuition of the link between network structure and stimulus encoding.

**Table 1:** Overview of the notation used in the present study (see Methods for details of the definitions).

〈 ⋅ 〉_*x*_	Average (over variable *x*)
*N*	Number of neurons
*r*(*s*)	Trial-averaged neural response (vector) to stimulus *s*
*C*	Covariance matrix across trials
$c_{ij}^{S},c_{ij}^{N}$	Pairwise signal/noise correlation coefficient
*c*_*S*_, *c*_*N*_	Population-averaged signal/noise correlation coefficient
*c*_in_	Input signal correlation coefficient
$\sigma_{\mu }^{2}$	Population variance projected on the average response
$\sigma_{d}^{2}$	Population variance projected on the diagonal direction
*d*	Vector along the diagonal direction
$\sigma_{all}^{2}$	Sum of neuron variances
*r*_ext_	Magnitude of external inputs (vector)
*V*_ext_	Variance of external inputs (vector)
〈*r*_ext_〉,〈*V*_ext_〉	Average over neurons (scalar)
*a*	Unobserved offset in firing rates
*G*	Matrix of direct recurrent interactions
*B*	Matrix of effective recurrent interactions
*F*	Matrix of feed-forward interactions
〈*B*〉,〈*F*〉	Average over matrix elements (scalar)
*ρ*	Variability of strength of interaction across neurons
*ρ*_ext_	Variability of external input across neurons
*S*	Signal-to-noise ratio for stimulus discrimination

**Table 2:** Summary of results on statistical signatures of three network scenarios. Average noise and signal correlation coefficients are denoted by *c*_*N*_ and *c*_*S*_, respectively, while *c*_in_ determines the strength of signal correlations in the input; see Methods. See S1 Appendix for detailed derivations.

Network model	〈*C*_*ii*_〉_*i*_	〈*C*_*ij*_〉_*i* ≠ *j*_	*c*_*N*_	*c*_*S*_
Recurrent network model	*N*〈*B*^2^〉(〈*r*〉(*s*) + *a* + 〈*V*_ext_〉)	*N*〈*B*^2^〉(〈*r*〉(*s*) + *a* + 〈*V*_ext_〉)	$\frac{1}{1+\rho }$	$\frac{1+\left(N-1\right)c_{\text{in}}}{1+\rho +\left(N-1\right)c_{\text{in}}}$
Feed-forward network model	〈*r*〉 + *a* + *N*〈*V*_ext_〉〈*F*^2^〉	*N*〈*F*〉^2^〈*V*_ext_〉(*s*)	$\frac{N{\langle F\rangle }^{2}\langle V_{\text{ext}}\rangle }{\langle r\rangle +a+N\langle V_{\text{ext}}\rangle \langle F^{2}\rangle }$	$\frac{1+\left(N-1\right)c_{\text{in}}}{1+\rho +\left(N-1\right)c_{\text{in}}}$
Gain-fluctuation model	$\frac{1}{N}V_{\text{ext}}\sum_{i}r_{i}^{2}+\langle r\rangle$	*V*_ext_〈*r*〉^2^	$\frac{V_{\text{ext}}{\langle r\rangle }^{2}}{\frac{1}{N}V_{\text{ext}}\sum_{i}r_{i}^{2}+\langle r\rangle }$	-

### Population signatures of noise statistics in the recurrent network model

**Fig 2:**
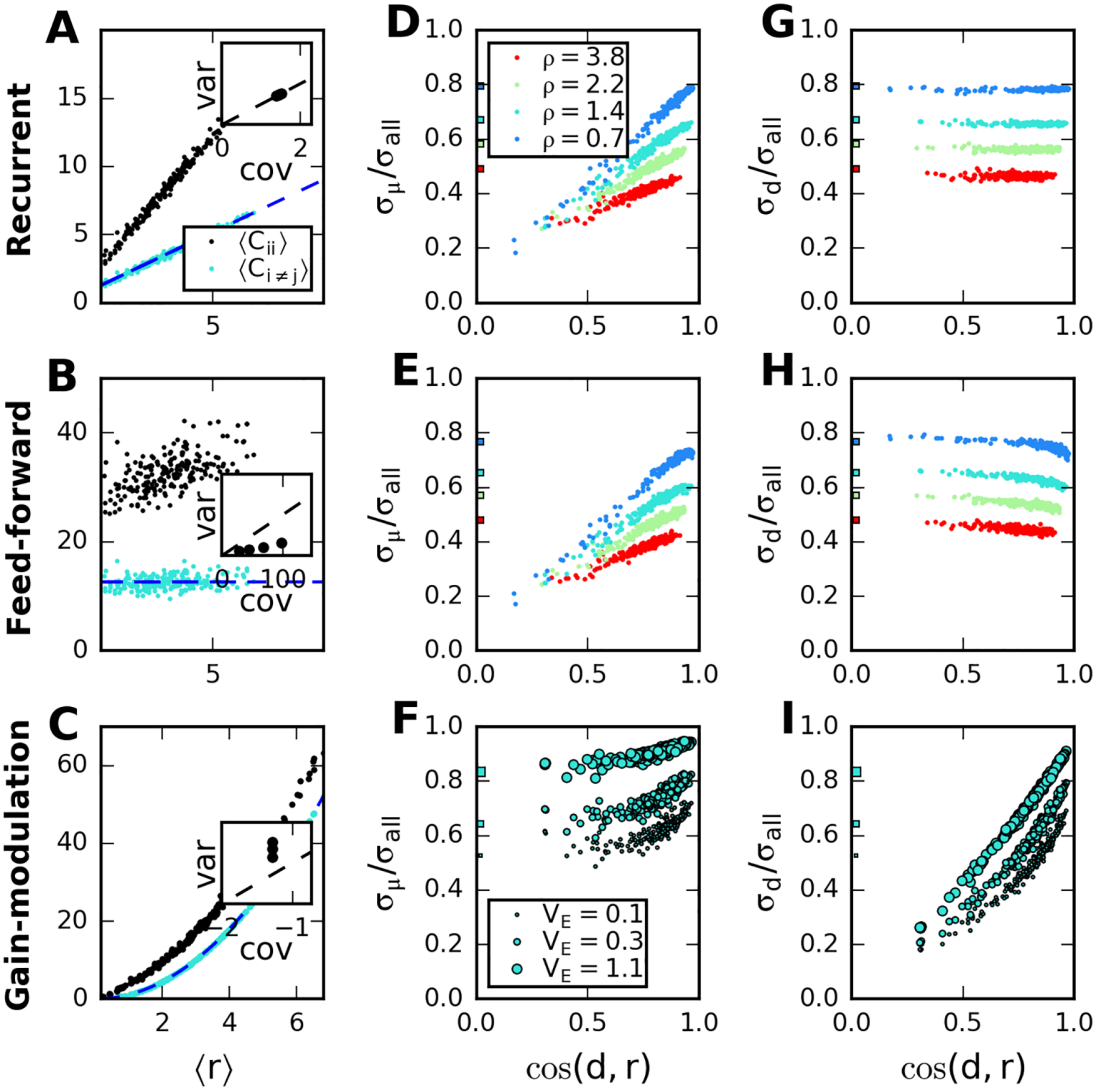
Response-covariance relations depend on the origin of correlations. A-C: Average population response, 〈*r*〉, versus average (co-)variances in recurrent network (top), feed-forward network with shared inputs (middle) and feed forward network with common gain fluctuations (bottom). Each dot corresponds to a random stimulus, blue dashed lines represent analytic results, Eqs (24)–(27). Larger *ρ* indicates larger variability of effective weights in the network models; larger *V*_ext_ indicates increased variance of gain fluctuations. Inset: Ratio slope/intercept of linear fits to the (co-)variances, for different networks; scatter plots of ratios for covariances vs variances show that they coincide only in the recurrent network model. D-F: Dependence of normalized variability projected on direction of average response for the three network architectures. In D, E, colors indicate results for different network parameters, in F, size of markers indicates strength of gain fluctuations. Square markers on the left indicate numerical value of $\sqrt{c_{N}}$. G-I: Same for the normalized variability projected on diagonal direction. See S1 Appendix for further details and the numerical parameters.

One population feature which distinguishes the possible mechanisms of generation of noise correlation is the relation between the population-averaged response, 〈*r*〉(*s*) = 〈*r*_*i*_(*s*)〉_*i*_ = ∑_*i*_ *r*_*i*_(*s*)/*N*, on the one hand, and the population-averaged variance, 〈*C*_*ii*_〉_*i*_(*s*) = ∑_*i*_ *C*_*ii*_(*s*)/*N*, or the noise covariance averaged across pairs, 〈*C*_*ij*_(*s*)〉_*i* ≠ *j*_ = ∑_*i* ≠ *j*_ *C*_*ij*_/*N*(*N* − 1), on the other hand. In this section, we analyze these relations for Poisson neurons in a recurrent network with random effective connections, as described in Methods. Using Eq (12) in which a baseline spiking rate has been included, which reads *C*_*ij*_ = ∑_*k*_ *B*_*ik*_ *B*_*jk*_(*r*_*k*_(*s*) + *a* + *V*_ext,*k*_) for the pairwise covariances, we derive the expression

$$\begin{array}{c} {\left\langle C_{ii}\right\rangle }_{i}\left(s\right)\approx N\left\langle B^{2}\right\rangle (\left\langle r\right\rangle \left(s\right)+a+\left\langle V_{\text{ext}}\right\rangle ) \end{array}$$ (23)

for the average variance, and the expression

$$\begin{array}{c} {\left\langle C_{ij}\right\rangle }_{i\neq j}\left(s\right)\approx N{\left\langle B\right\rangle }^{2}(\left\langle r\right\rangle \left(s\right)+a+\left\langle V_{\text{ext}}\right\rangle ) \end{array}$$ (24)

for the average covariance. (See S1 Appendix for mathematical details and Fig 2 for numerical results.) The quantity 〈*B*〉 = ∑_*ij*_ *B*_*ij*_/*N*^2^ is the average strength of the effective connections in neuron pairs and, correspondingly, $\langle B^{2}\rangle =\sum_{ij}B_{ij}^{2}/N^{2}$ is the average of its square. The variance of the input, averaged across neurons, is 〈*V*_ext_〉, and *a* denotes a possible constant offset in the observed firing rates (firing rate baseline).

Eq (24) yields a linear relation between the average spiking rate and the covariances. The factor of proportionality—the ‘slope’—has been derived, here, with the assumption that the elements of *B* are independent. If this is not the case, the slope will also depend on the correlations among the matrix the elements, *B*_*ij*_. However, the linear dependence between rates and (co-)variances itself, which results from the nature of the Poisson spike generation, is independent of this assumption. In the linear relations between average variance and spiking rate, and between average covariance and spiking rate, the ratio of the intercept and the slope is identical and equal to the strength of the external noise. In summary, in the recurrent network model, both internally generated noise and external noise are amplified (or attenuated) by the recurrent connections, and the parameters in the relations between (co-)variances and population response can be related to network and input parameters.

### Population signatures of noise statistics in the feed-forward and gain-modulation network models

In feed-forward networks, both shared input units and global gain fluctuations can yield noise correlations [7, 9, 13]. Here, we compare the population signatures of the noise statistics in the case of these two alternatives with those that emerge in a recurrent network model.

In a feed-forward network, pairwise covariances are given by Eq (17), which can be rewritten as

$$\begin{array}{c} C_{ij}=\delta_{ij}\left(r_{i}+a\right)+\sum_{k}F_{ik}F_{jk}V_{\text{ext},k}. \end{array}$$ (25)

Note that, in contrast to the case of a recurrent network, the neural firing rates, *r*_*i*_, only affect diagonal entries, i.e., variances. The average covariance can be expressed as

$$\begin{array}{c} {\left\langle C_{ij}\right\rangle }_{i\neq j}\left(s\right)\approx N{\left\langle F\right\rangle }^{2}\left\langle V_{\text{ext}}\right\rangle \left(s\right), \end{array}$$ (26)

and does not directly depend on the average population response, 〈*r*〉(*s*). The relation between the average input variance, 〈*V*_ext_〉, and the average population response depends upon on the balance between positive and negative elements in *r*_ext_, which we measure through the input variability, *ρ*_ext_ = *var*(*r*_ext_)/〈*r*_ext_〉^2^ (see S1 Appendix).

In a model network with global gain fluctuations, the average covariances scale quadratically with the population response, as

$$\begin{array}{c} {\left\langle C_{ij}\right\rangle }_{i\neq j}=V_{\text{ext}}{\left\langle r\right\rangle }^{2}. \end{array}$$ (27)

This is a direct consequence of the fact that pairwise covariances are proportional to the product of the firing rates of the two neurons in the pair. By contrast, the single-cell variances and the population-averaged variance each have both quadratic and linear contribution in *r* and 〈*r*〉, respectively (see also e.g. [9]). (Further details are provided in S1 Appendix.)

Additional discrepancies between the different scenarios can be related to the detailed structure of the matrix *C*, which determines the shape of the response distribution. By ‘shape’ we refer to the geometric orientation and extent of the multi-dimensional ellipsoid cloud that corresponds to the population responses, in the space spanned by the responses of the individual neurons. A two-dimensional sketch of such an ellipse, and the geometric interpretation of the quantities we use, are depicted in Fig 1A (see also Methods). Loosely speaking, the orientation of the response distribution depends on the network architecture: the long axis of the ellipsoid, i.e., the direction of the largest variance, lies along the diagonal in the cases of the recurrent network model and the feed-forward model with shared inputs, but instead along the direction of the mean response in the case of the gain-fluctuation model (Fig 1B). (The “diagonal direction” corresponds to a population response pattern in which all neurons are equally active.)

To capture this dependence quantitatively, we consider the projected variances, $\sigma_{\mu }^{2}$ and $\sigma_{d}^{2}$ (see Fig 2, and Methods for details). Apart from differences in the relations between population-averaged response and variance, Fig 2A–2C, we find that, in the gain fluctuation model, but not in the other two models, the variance projected along the direction of the average response is approximately constant across stimuli (Fig 2D–2F). In recurrent and feed-forward network models in which the connections induce strong correlations, a large proportion of the variance lies along the diagonal direction, independently of the stimulus, in contrast to the outcome of the gain-fluctuation model (Fig 2G–2I). The variance projected along the direction of the average response is large only if the latter happens to be similar to the diagonal direction. For a given data set, a plot of the relations between population response and (co)variance can thus be used as a simple test for the consistency of the data with the different circuit models.

### Relation between signal correlation and noise correlation in the recurrent network model

**Fig 3:**
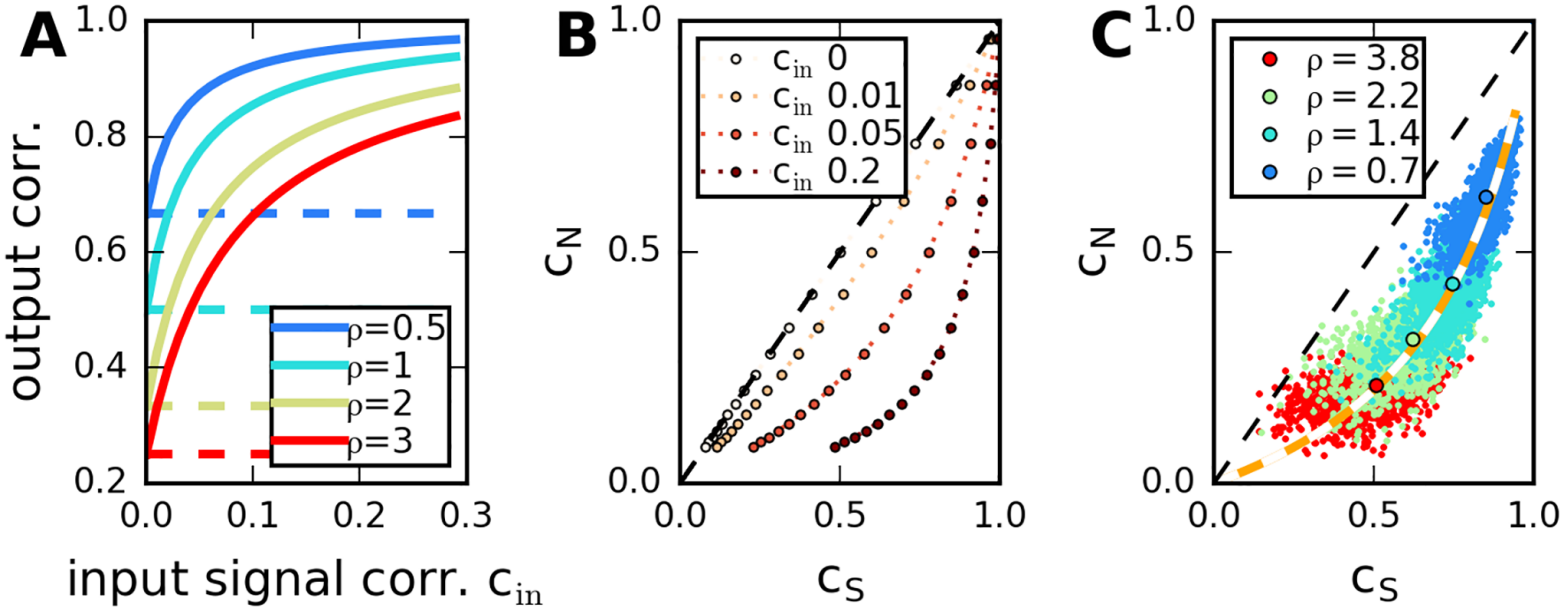
Relation between signal and noise correlations in the recurrent network model. A: Dependence of average signal correlations, *c*_*S*_ (continuous lines), and average noise correlations, *c*_*N*_ (dashed lines), on input correlation and network properties, Eqs (28) and (29). Low variability of the transfer matrix elements, *ρ*, increases *c*_*N*_ and *c*_*S*_. Input signal correlations affect only *c*_*S*_. B: Same data, average signal versus noise correlation. C: Scatter plot of all pairwise signal versus pairwise noise correlations (dots), in five network realizations, for *c*_in_ = 0.05. Circles indicate network average across pairs, orange dotted line corresponds to analytical expressions displayed in B for *c*_*N*_ and *c*_*S*_.

In the simple scenarios we analyze here, average responses and covariances are related because they both depend on the network architecture. By expressing signal correlations, $c_{ij}^{S}$, and noise correlations, $c_{ij}^{N}$, in terms of network and input parameters, we can derive a relation between these two sets of quantities (Fig 3). For a random transfer matrix, *B*, all neuron pairs are statistically equivalent, and the strength of correlations can be characterized by the average of the pairwise signal and noise correlations. In a recurrent network model obtained from a random matrix *B* in which elements are independent, the network architecture affects noise and signal correlations through an effective quantity, namely, the signal-to-noise ratio of the elements of the transfer matrix, *ρ* = *var*(*B*)/〈*B*〉^2^:

$$\begin{array}{c} c_{N}\equiv {\left\langle c_{ij}^{N}\right\rangle }_{i\neq j}\approx \frac{1}{1+\rho }, \end{array}$$ (28)

$$\begin{array}{c} c_{S}\equiv {\left\langle c_{ij}^{S}\right\rangle }_{i\neq j}\approx \frac{1+\left(N-1\right)c_{\text{in}}}{1+\rho +\left(N-1\right)c_{\text{in}}} \end{array}$$ (29)

(see S1 Appendix, Eqs (19) and (26)). Here, *c*_in_ denotes the strength of signal correlations in the input across stimuli. Both signal and noise correlations are larger in networks with more homogeneous entries (smaller *ρ*).

If the average input for pairs of neurons across stimuli is uncorrelated (no input signal correlations, *c*_in_ = 0), noise and signal correlations are identical on average. However, due to a prefactor which grows with system size (*N* − 1 in Eq (29)), even weak input signal correlations are strongly amplified and can yield a large effect, Fig 3A. Because noise in the population response to a given stimulus is produced internally in the process of spike generation, noise covariances are unaffected by this mechanism, so that strong signal correlations can coexist with weak noise correlations, Fig 3B. These relations depend on network parameters and hold for the average correlations (across pairs in a network). The pairwise noise and signal correlation coefficients, $c_{ij}^{N}$ and $c_{ij}^{2}$, can vary widely, but numerical calculations indicate that they are correlated (across pairs) in a given network, Fig 3C. For the corresponding relations in the other two models, see Table 2. Details of the derivations are reported in S1 Appendix, as well as in previous work on the gain-fluctuation model (see Discussion).

### Stimulus discrimination in the recurrent network model

**Fig 4:**
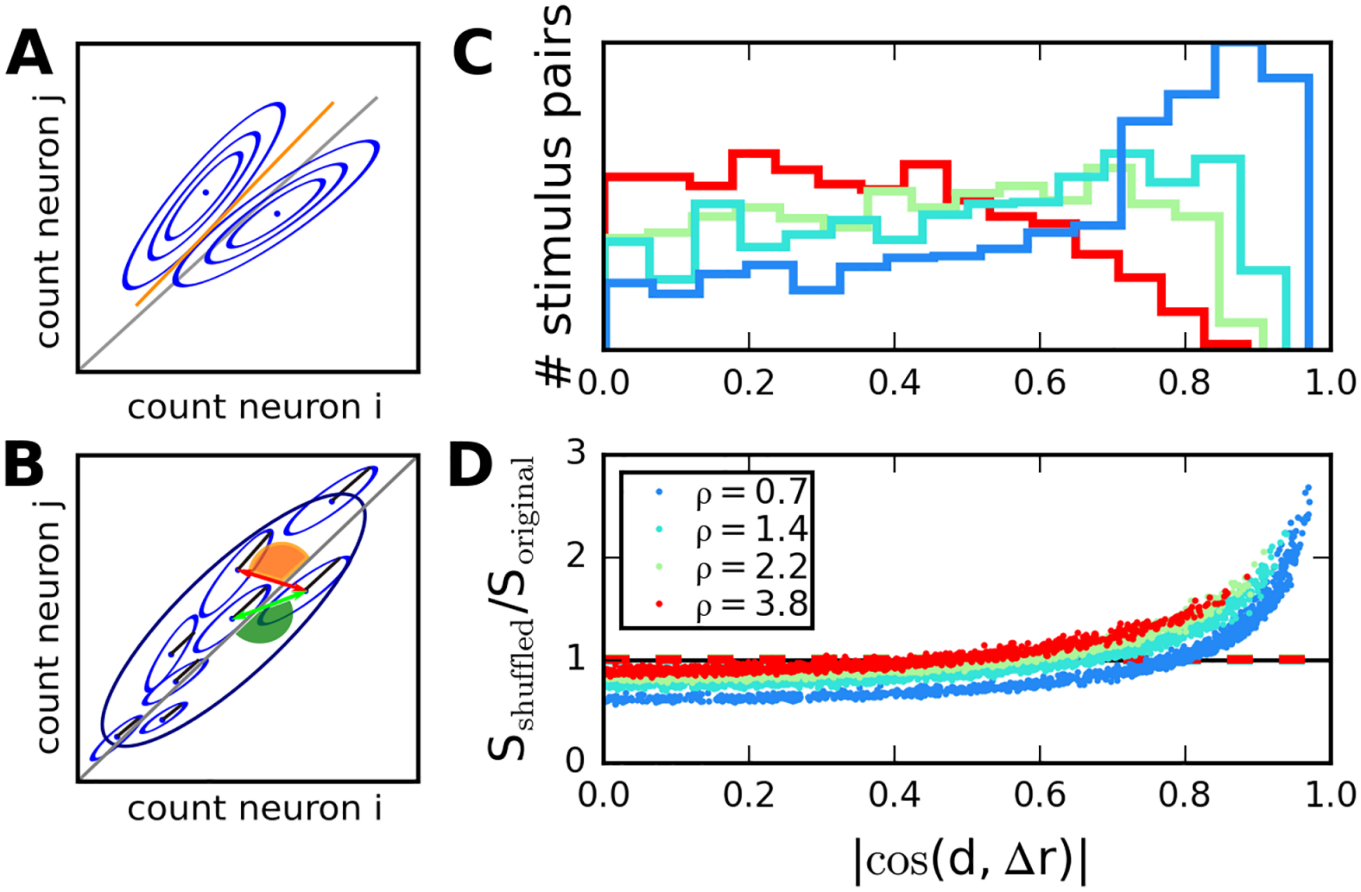
Covariances affect stimulus discriminability. A: Sketch of response distributions for two neurons and two stimuli (blue ellipses). Neural responses are correlated for both stimuli, so that main axis of variability is nearly parallel to linear separator (orange line). B: Larger ensemble of stimuli for correlated neurons (large variance along diagonal directions). The effect of correlations is favorable for stimulus pairs for which difference in means is orthogonal to diagonal (red arrow), and unfavorable when difference in means is nearly parallel to diagonal (green arrow). C: Distribution of cosines of angle between diagonal and difference in means, across stimulus pairs in recurrent networks with different parameter *ρ*. In networks with low *ρ*, unfavorable angles are more frequent. D: Ratio between discriminability for correlated and shuffled distributions, dots correspond to stimulus pairs. Values smaller than one correspond to beneficial correlations. Dashed horizontal lines indicate averages across all stimulus pairs.

Since the network architecture affects both signal and noise correlations, it is natural to ask how the discriminability of stimuli is affected in turn, as it depends on both quantities. Indeed, noise correlations can affect the coding properties of a population appreciably [24]. Noise correlations are referred to as favorable for the discrimination of a pair of stimuli when the shapes of the two correlated response distributions (corresponding to the two stimuli) are such that variability occurs predominantly in a direction orthogonal to the informative direction (Fig 4). The relevance of correlations can be quantified by comparing the discriminability of stimulus pairs in the case of the full population output and in the case in which noise correlations are removed by shuffling trials.

A number of earlier studies have considered the influence of correlated variability on coding [23, 26, 28, 29, 32]. Most of these, however, did not consider the dynamical or mechanistic origins of correlations. Some recent investigations have related coding with correlated variability to questions of dynamics and circuits [7, 9, 11, 13, 14], but these have focused on feed-forward architecture and, primarily, small populations of neurons. By contrast, we consider here larger, recurrent networks.

In Fig 4, we analyze, in the recurrent network model, the interplay of signal and noise correlations that influences stimulus discrimination. A dominant portion of the variance occurs along the diagonal (Fig 4A), so that stimulus discriminability depends on the angle between the diagonal and the line connecting the two average population responses (corresponding to the two stimuli). In the case of a family of stimuli (Fig 4B), discriminability depends on the distribution of the many angles corresponding to different choices of pairs of stimuli. Here, we illustrate this quantity (Fig 4C), and we compare discriminability in the recurrent model to that for independent neurons (Fig 4D), in the case of a population with 60 neurons. We find that the more heterogeneous our recurrent connections, i.e., the larger the parameter *ρ*, the more frequently small cosines, i.e., favorable angles, occur (Fig 4C). We then compare discriminability (through the signal-to-noise ratio, *S*, defined in Eq (22) in the recurrent network model with that in a population of independent neurons obtained by shuffling across trials (Fig 4D). We find that the effect of shuffling depends on network heterogeneity as well (via the distribution of the noise correlations); however, when averaged over all pairs of stimuli, the effect of correlations is negligible and does not depend on the network’s heterogeneity. In the case of more homogeneous recurrent networks, both the beneficial and harmful effect of noise correlations are boosted, for different stimulus pairs. We note that similar effects can be observed in the model of feed-forward network with shared input and in the model of feed-forward network with gain fluctuation; see S1 Fig.

In S2 Appendix we analyze idealized two- and four-population models of recurrent networks which provide further intuition on the relation between network structure and the representation of stimulus information. We illustrate the way in which the accuracy of stimulus representation depends on the synaptic strengths; among other things we show that, by adequately varying the within-population and cross-population synaptic strengths, stimulus representation can improve while firing rates and variance remain unchanged. Intriguingly, this improvement is accompanied by an increase of the noise entropy in the network, rather than the more commonly expected decrease of the noise entropy.

### Variability and correlations in populations of mouse cortical auditory neurons

We use the theoretical results presented in the previous sections to analyze the responses of populations of neurons to different stimuli. The data set contains the firing rates, collected during a certain time interval, in response to the presentation of different sound stimuli in a number of trials (see Methods and Ref. [35]). We will compare the properties of covariances (across trials) and average responses with the predictions of our different models. Based on the models, we can then evaluate the effect of network-generated variability on stimulus discrimination.

**Fig 5:**
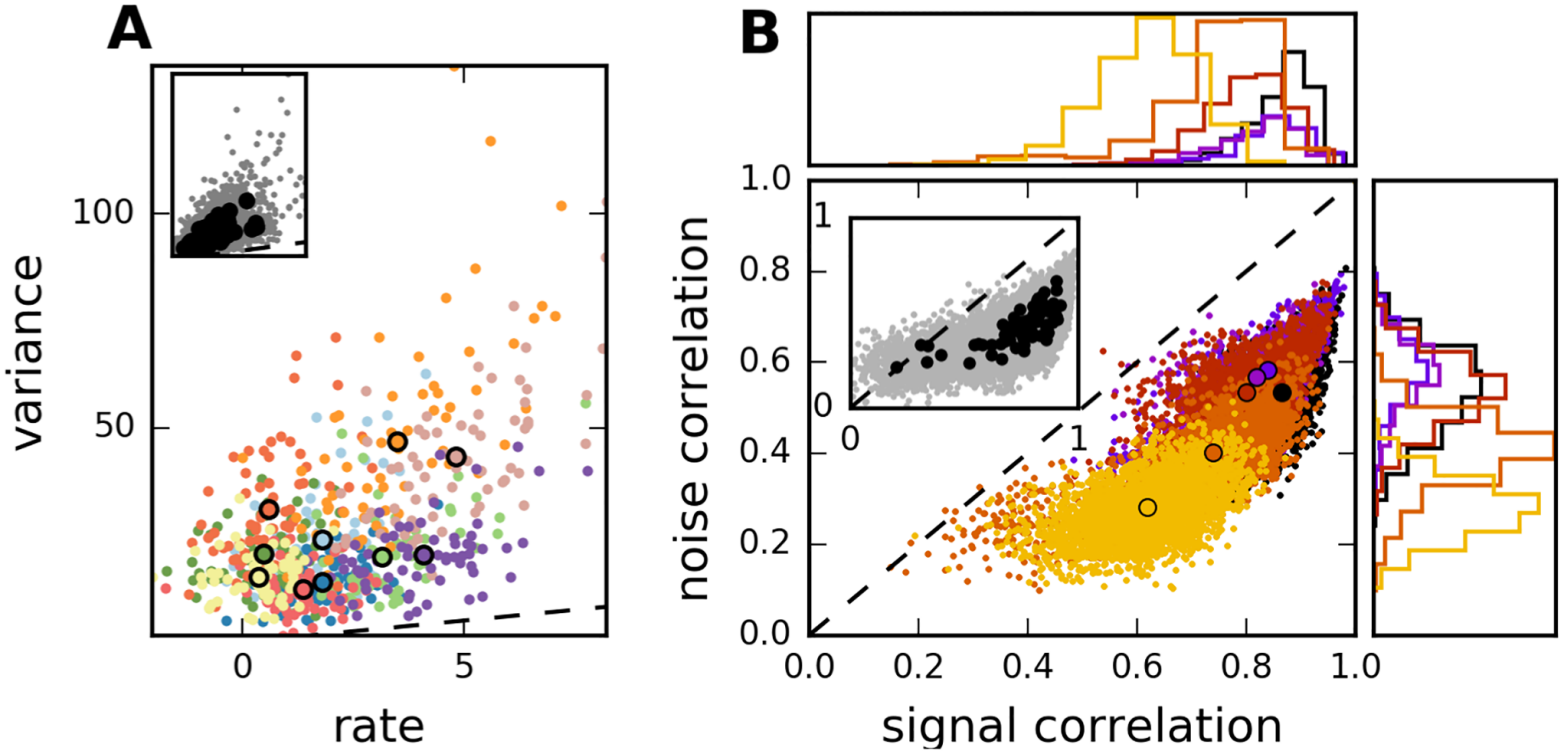
Variability and correlation in mouse auditory cortex. A: Average response versus response variance in a single population. Colors correspond to 10 randomly chosen stimuli. Dots correspond to single neurons, circles to averaged values across population. Dashed line indicates identity. Inset: All neurons for all stimuli. B: Scatter plot of signal correlation coefficient (across stimuli) vs. noise correlation coefficient (averaged over stimuli), for different neural populations measured in the same animal; marginal histograms at top and bottom. Signal and noise correlations are correlated across pairs. Correlations are high in general, but the amount of signal and noise correlations varies strongly across populations. Circles denote average across pairs in each populations. Inset: Black circles, average signal and noise correlations in all measured populations. Grey dots, individual pairs, 5% randomly chosen from all experiments.

The trial-to-trial variability in single-neuron output is large (supra-Poisson, Fig 5A); neurons with larger average response exhibit also a larger variability in their responses. This tendency is observed across different neurons responding to a given stimulus, for individual neurons across stimuli, and for the average population response across stimuli. The pairwise correlations in trial-to-trial variability are quantitatively large and pairs with high noise correlations tend to have strong signal correlations, Fig 5B. In other words, because signal correlations measure the similarity of average responses across different stimuli, neurons with similar tuning properties also have similar trial-to-trial variability. Additionally, in populations with strong average signal correlations, noise correlations are strong as well. These relations between noise and signal correlations can be reproduced in the recurrent network model (Fig 3). However, similar results may be possible also in alternative scenarios for the generation of correlated variability. We compare the different models in the next section.

### Analysis of population response variability in mouse auditory cortex

**Fig 6:**
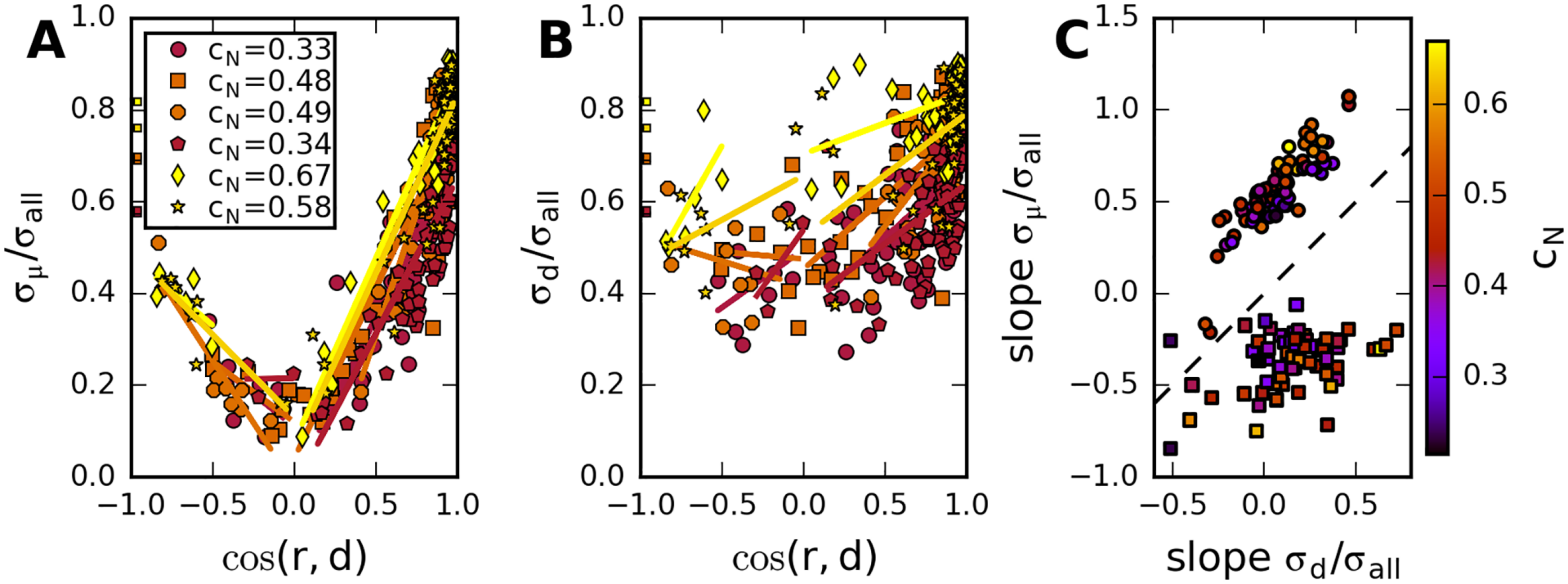
Dependence of noise distribution orientation on average response, in data. A, B: Typical examples, 6 neural populations recorded in the same animal. Relative variances projected on mean and diagonal directions, versus cosine of angle between mean response and diagonal. Each marker corresponds to a different stimulus. Different markers/colors denote different populations. Squares to the left side indicate $\sqrt{{\langle C_{ij}\rangle }_{i\neq j}/{\langle C_{ii}\rangle }_{i}}$. Solid lines: linear fits. C: Slopes from linear fits as in A, B of *σ*_*μ*_/*σ*_*all*_ vs. slope from *σ*_*d*_/*σ*_*all*_, for all measured populations. Circles correspond to slopes for positive, squares to negative cos(*r*,*d*). Colors in all panels indicate value of *c*_*N*_ in populations.

Motivated by our investigation of network models, we first examine the relation between the shape of the response distributions for each stimulus and the pattern of average responses for the entire set of stimuli. In Fig 6A and 6B, we display the normalized standard deviation projected on the direction of the mean response, *σ*_*μ*_/*σ*_*all*_, and the normalized standard deviation projected on the diagonal direction, *σ*_*d*_/*σ*_*all*_, as functions of the angle between average response and diagonal, represented as cos(*d*, *r*) (see Methods for the definitions of these quantities). We observe a strong dependence on cos(*d*,*r*) of the variance projected on the direction of the mean response, but not of the variance projected on the diagonal direction. The dependence of *σ*_*μ*_/*σ*_*all*_ on the stimulus is much stronger in nearly all the measured populations, Fig 6C. This behavior is consistent with a network model, either feed-forward or recurrent, but not with a model with shared gain fluctuations, where a large part of the variance is consistently in the direction of the mean response (see Sec. “Population signatures of noise statistics in feed-forward network models”). We note, however, that in the data firing rates are measured relative to the spontaneous activity; this is reflected in the presence of negative values in the stimulus-evoked activity. Such an offset could affect our comparison because we do not know the true value of the mean response. To account for this possibility, we searched for the best possible offset, assuming the model of gain fluctuations for the stimulus-dependent covariances. We then corrected the firing rates by this offset, and evaluated the stimulus dependence of *σ*_*μ*_ and *σ*_*d*_, as before (see S1 Appendix for details), but found no qualitative change in the results. However, we did not test for mixed models, and it is possible that part of the correlated variability can be explained by common gain fluctuations.

Based on the results presented above, we conclude that, of our three scenarios, feed-forward and recurrent network models are more consistent with the data. In the following, we fit the parameters of these models to the data, to find which of these two provides a better fit. For this comparison, we analyze the dependence of the average variances, 〈*C*_*ij*_〉_*i* ≠ *j*_, and covariances, 〈*C*_*ii*_〉_*i*_, on the population-averaged response, 〈*r*〉(*s*). In the recurrent network (Eqs (23) and (24)), both variances and covariances increase linearly with 〈*r*〉, while in the feed-forward network (Eq (27)), mean covariances are not expected to depend strongly on 〈*r*〉.

**Fig 7:**
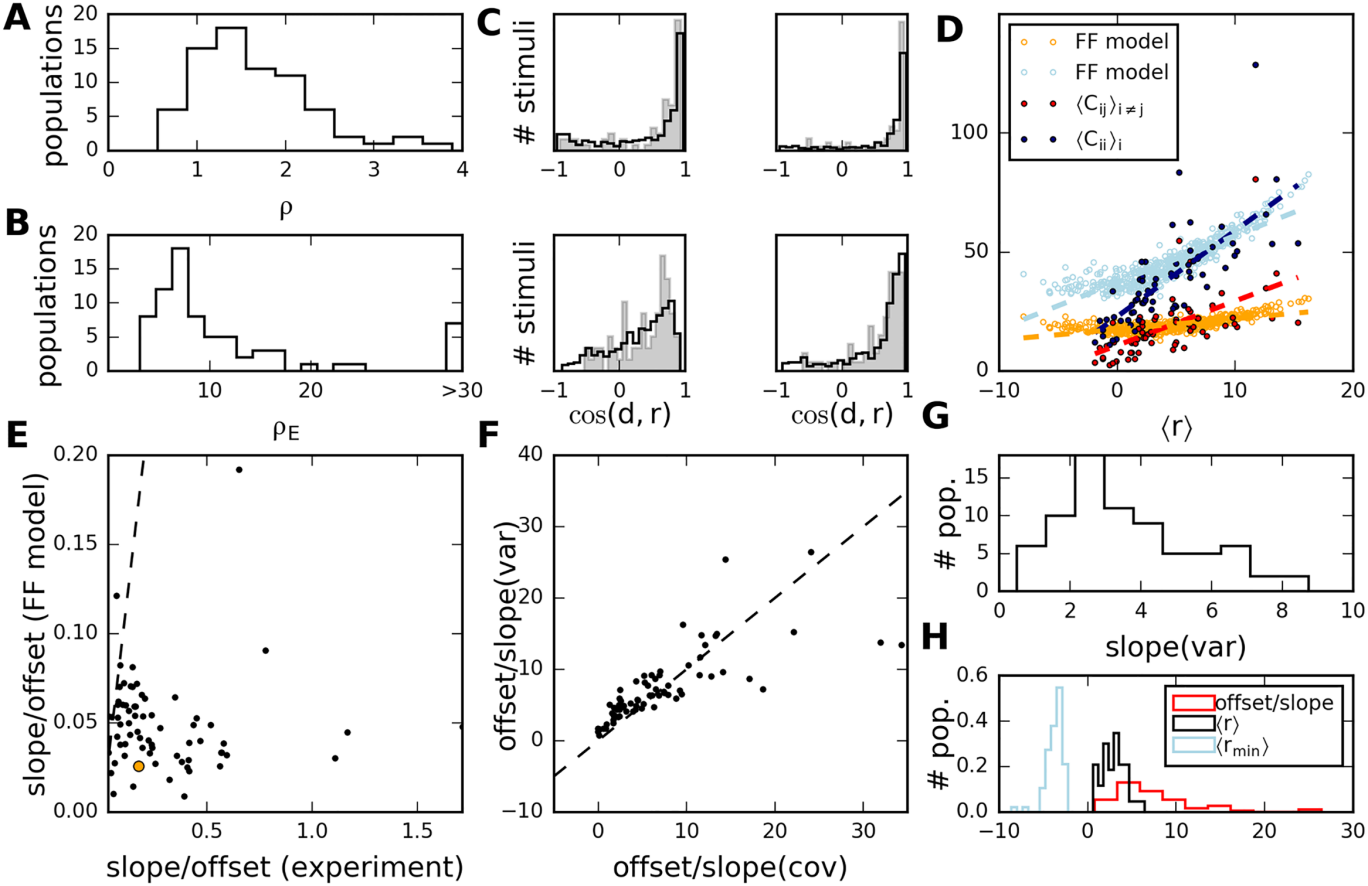
Scaling of covariances with average response, in experiments and models. A, B: Distribution of parameters *ρ* and *ρ*_ext_ (variability of network connections and stimulus input) estimated from all experiments. C: Histograms of cos(*d*,*r*(*s*)) from mean responses across stimuli, for a selection of populations recorded from in one animal. Filled histograms: experimental data. Solid lines: model results for 500 randomly generated stimuli and one network with random effective connections, parameters inferred from data. D: Population-averaged variances and covariances versus population-averaged response, for all stimuli (full circles) in one experiment and for corresponding feed-forward models (empty circles). Dashed lines represent linear fits. E: Scatter plot of ratio slope/intercept from linear fits in all experiments (excluding four outliers with very low intercept) versus corresponding values in the feed-forward model. Orange circle indicates population used in panel D. F: The ratio intercept/slope is consistent for covariances and variances, across experiments (dots). G: Distribution of estimated slopes for average variances. H: Distribution of estimated 〈*V*_ext_〉 (intercept/slope from fits to variances) compared to distribution of average and minimum firing rate across experiments.

To examine whether the experimental data is consistent with the feed-forward model, we fitted parameters to the activity of each experimentally observed population (see S1 Appendix), and generated a surrogate set of responses and covariance matrices, with a matching number of neurons and stimuli. We followed this procedure because, here and in the following, we do not fit a full connectivity matrix, but generate a matrix, *F*, with random entries from a distribution with parameters to fit the data at the population level. In particular, we obtained values for the variability in the input ensemble, *ρ*_ext_ = *var*(*r*_ext_)/〈*r*_ext_〉^2^, and in the network connection strengths, *ρ* = *var*(*F*)/〈*F*〉^2^. We found that *ρ*_ext_,*ρ* > 1: for both input and network elements, the variance was (appreciably) larger than the mean (see Fig 7A and 7B). A large value of *ρ*_ext_ is needed to explain the high variability of average responses across stimuli, while the variability of the network connections captured by the parameter *ρ* is related to the magnitude of the observed correlations. With these parameters, the statistics of the distribution of average responses are well reproduced (Fig 7C). As mentioned in Sec. “Population signatures of noise statistics in feed-forward network models”, for large values of *ρ*_ext_ the feed-forward model does not predict a strong dependence of 〈*C*_*ij*_〉_*i* ≠ *j*_ on 〈*r*〉(*s*). We quantify this relation by the ratio of slope to intercept in a linear fit of the data; this ratio measures the strength of dependence of average covariances on population responses.

In the feed-forward scenario, we find that the slopes of the linear fits in the model relative to the intercepts are too low in comparison to what is observed in the experiments (Fig 7D and 7E). The feed-forward model cannot reproduce both the large variability of neural responses across stimuli and the increase of the average covariance with the average response. In a recurrent model, this increase is expected, because noise generated by the neurons is propagated through the network: the covariances are proportional to the average response (see Eq (24)). Moreover, it predicts that the ratios of intercept and slope in the linear behaviors of variances and covariances are the same (this ratio scales with the variance of the external input). Indeed, linear fits to these relationships reveal approximately consistent ratios of intercept and slope (panel F); the estimated parameters are summarized in Fig 7G and 7H. The external noise, estimated from the ratio intercept/slope, turns out to be of the same magnitude as the average rate. Interpreted in terms of the model, the noise resulting from Poisson spike generation thus contributes as much to neuron variance as the external input. This combined variability is propagated through the network, and, on average, multiplied by a factor *N*〈*B*〉^2^ > 1, corresponding to the slope in Eq (24). If this factor is larger than one, both average response and noise are amplified by the recurrent connections.

In summary, we find that a model of recurrently connected neurons with random effective connections captures the observed activity statistics, in particular the relations between average response and average covariances as well as the consistent shape of population fluctuations across stimuli. Feed-forward networks models with either shared input or common gain fluctuation are not consistent with all of these observations. We note, however, that our conclusions are based on a relatively small data set; a larger number of trials and measurements of absolute values of firing rates would be desirable for a more stringent test.

### Influence of noise correlation on stimulus coding in mouse auditory cortex

**Fig 8:**
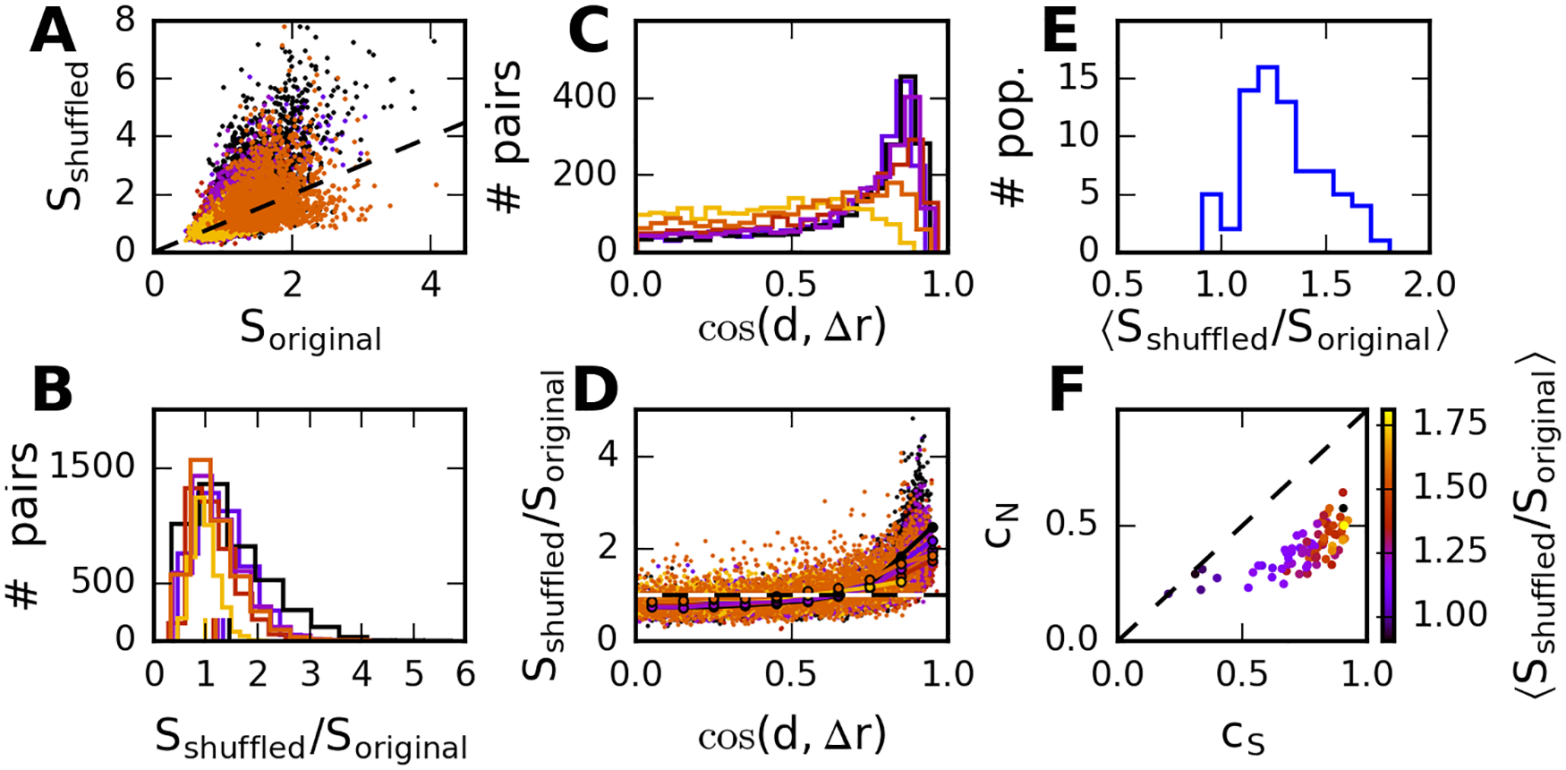
Effect of correlations on stimulus discrimination. A: Signal-to-noise ratio *S*, dots correspond to pairs of stimuli. Value from observed covariance matrix versus value based on shuffled trials, for different populations (different colors) in the same animal. B: Same data, distribution of *S*(shuffled)/*S*(original). Broad distribution, but mean value greater than one for all populations (tics) indicates that shuffling increases information on average. C: Distribution of cosine of angle between diagonal and average-response difference, across stimulus pairs for different neural populations. Large cosines/small angles are most frequent. D: Scatter plot of effect of correlations on discrimination. Small dots correspond to stimulus pairs. Connected large dots indicate average value in a bin centered at the corresponding location on the x-axes. E: The distribution of mean values across all measured populations shows that correlations are on average unfavorable, for almost all populations. F: Average effect of noise correlations is stronger for larger signal correlations.

We quantify the influence of noise correlation on stimulus discrimination by the ratio *S*_shuffled_/*S*_original_, where the signal-to-noise ratio, *S*, is calculated for the data before and after shuffling trials. The quantity *S* is defined in Eq (22) and denotes, for a pair of stimuli, the difference in average response divided by the standard deviation of the responses, both projected on the most discriminant direction. Larger values indicate that stimuli are easier to discriminate; if *S*_shuffled_/*S*_original_ is larger than one, then removing correlations by shuffling trials improves stimulus discrimination. Across pairs of stimuli, this signal-to-noise ratio varies strongly in the data (Fig 8A). On average, stimuli are slightly easier to discriminate in the shuffled data, i.e., noise correlations are weakly harmful to the coding of the stimulus set used in the experiments Fig 8B.

A closer analysis of the response distributions reveals that, to a large degree, the effect of shuffling can be explained by the relative locations of the two mean responses with respect to the diagonal, Fig 8C and 8D, as measured by cos(*d*,*r*(*s*_1_) − *r*(*s*_2_)) (Fig 4). This is because, due to the noise correlations, the main direction of variability is along the diagonal. The overall effect of shuffling on stimulus discrimination depends on the relation between noise and signal correlations, Fig 8E and 8F: stronger signal correlations lead to a stronger dominance of angles that are unfavorable if noise is aligned along the diagonal direction, and in this case shuffling correlations away will benefit stimulus discrimination.

In the above, we investigated the influence of noise correlation on stimulus coding. A more biologically relevant question pertains, rather, to the influence of circuit architecture and parameters on coding. These govern the response statistics, which in turn influence the coding performance. Upon varying circuit properties, in general both average responses and their higher-order statistics can change [18, 30]. The overall effect of the network stimulus information during this transformation of the input into the spiking output depends on the noise generated intrinsically—by the Poisson spike-generation mechanism. Noise in the input-output transformation necessarily destroys some of the information contained in the input, so that this transformation is lossy. Stronger excitatory connections generically amplify both average responses and the trial-to-trial variability. Specifically, the variance of Poisson neurons scales with firing rate. In a feed-forward network, this is the only source of noise, and the input to the neurons increases with the strength of the feed-forward connections. As a result, the signal-to-noise ratio improves with stronger excitatory connections: the enhancement in the firing rates overcompensate the increase in noise. By contrast, in a recurrent network, noise from Poisson spike-generation is fed back into the network and this causes fluctuations of the firing rates themselves. These fluctuations are also amplified by the recurrent connections, and consequently the signal-to-noise ratio decays with stronger excitatory connections. Formally, the distinction between feed-forward and recurrent networks is embodied in dependence of the (co)variances on network parameters and average responses (Eqs 17 and 12).

To examine the combined effects of recurrent connections on average responses as well as variability around these and its correlation, it is instructive to consider a simplified, recurrent network model; we discuss such a model in detail in S2 Appendix. While the theoretical framework is not a new one, the advantage of this simple model is that the effect of recurrent connections can be calculated explicitly. We show that, provided the recurrent connections are sufficiently strong, shuffling correlations will reduce stimulus discriminability. This effect arises because the shuffling removes the effect connections have on correlations, but not their effect on the average responses and single cell variances.

## Discussion

The relation between circuit architecture and the representation of information in neural populations is central to quantitative neuroscience. And, in particular, the relation between circuit architecture, population response fluctuation, and coding is the object of much study [8]. In the present work, we seek to understand the influence of correlated variability on stimulus representation, based on the exploration of model networks of stochastic neurons together with data analysis. We modeled neural activity using a set of coupled Poisson processes in order to study the relations between covariances and average responses in a neural population, across different stimuli. We found that these relations differ depending on the network architecture, and that correlations observed in mouse auditory cortex are consistent with those generated in a recurrent network model. This model also helps us interpret the effect of the observed correlations on the discriminability of stimuli and the nature of the propagation of information through the network.

### Modeling the structure of correlations in neural circuits

We studied correlated variability theoretically using the idealized framework of Poisson neurons and random effective networks. These simplifications allow us to focus on qualitative differences arising from different network architectures; in turn, the identified qualitative trends can be tested against experimental data without having to infer the detailed connectivity structure. On the down side, we cannot fit individual pairwise correlations. Also missing is a thorough investigation of the effects of more biologically realistic, non-random connections. In particular, specific connectivity structures which may give rise to different response regimes [40], such as weakly correlated activity, remain to be investigated.

A broad range of quantitative work has been devoted to examining the origins of variability in the activity of neural populations. Recurrence has been invoked to explain population variability in the absence of microscopic stochasticity [41, 42], and more recent work along has extended this line of thought to cover a greater range of response regimes of asynchronous [43–45]. Population-wide fluctuations have been identified as contributing strongly to the observed correlations between the responses of cortical neurons to sensory stimuli [10]. Global, multiplicative gain fluctuation has been proposed also as a mechanism for the generation of such correlations [11], and its implication on the form of the correlation has been analyzed and fitted successfully to cortical data [9]. Similar models capture noise correlations, and their stimulus dependence, in the retina [13, 14], as well as the large correlations observed under anesthesia [12]. Finally, global gain fluctuation models have also been used to reproduce the relations between noise and signal correlations [7].

In general, it is difficult to differentiate the mechanism of shared input from that of common gain fluctuation, and even from that of recurrence, as the origin of correlated variability, based on measured activity. Here, instead of focusing on temporal dynamics [16, 46, 47], we approach the problem by considering the relation between the network architecture and the structure of population activity statistics, when a neural population is presented with an ensemble of stimuli. In principle, the observation of population responses to a battery of stimuli provides sufficiently many constraints to tease apart different network models, and infer the parameters in the corresponding connectivity matrices. In practice, and especially for large neural populations, the number of trials accessible in experiments constrains the precision of measured covariances and the resolution of the fits. Here, we took a macroscopic view, by considering measurable effects at the population level, rather than aiming to estimate the weights of individual (effective) connections. As such, we compare population quantities derived from our models to population quantities extracted from the data, rather than to individual, pairwise correlations or other such microscopic quantities.

Our theoretical analyses approximate the spike generation mechanism in neurons by a Poisson process; as such, single-cell variability scales with the spiking rate. If the noise generated within the network were purely additive, and, hence, independent of spiking rates, it would be indistinguishable from external noise that is simply filtered through the network [38]. In the Poisson model, by contrast, the interplay of internally generated noise and variability due to external signals imprints its signature on the dependence of covariances on firing rates. Even if Poisson variability happens to be a faithful model of single-cell variability, still our models make the limiting assumption of linearly interacting Poisson processes. Clearly, nonlinear transfer functions can affect the relation between spiking rates and noise correlations in individual pairs of neurons [8, 48]. The tractability of the model, however, allows us to obtain explicit relations between observable statistics, which can be used as a starting point to interpret the origin of correlated variability.

### Influence of correlations on stimulus discrimination in neural populations

We used a recurrent network with random effective connections to estimate the influence of the variability generated in the network dynamics on stimulus representation. Based on the paradigm that response variability amounts to noise that downstream neurons have to cope with, a number of authors have argued that noise correlations can benefit information coding, provided they suppress variability along the direction relevant for stimulus discrimination [24–26, 29]. The effects of additive and multiplicative origins of noise correlation have been examined in Refs. [11, 13, 14]. As in earlier work, we quantified the effect of noise correlation on coding by comparing the true or model data with their modified versions obtained from shuffling trials: this allows for a comparison between the correlated population and a mean- and variance-matched independent population. In our work, however, we compared the outcome of this procedure in the case of three different network architectures, and in the context of large populations of (model) neurons presented with a battery of stimuli.

Recent theoretical studies have examined the impact of noise and correlations generated in recurrent networks. Noise internally generated in a network via spike generation can in principle be averaged out in feed-forward or recurrent networks, depending on the connectivity of the network, by increasing the population size and the redundancy of the population code [30]. However, external noise and ‘sub-optimal processing’ [33] can limit the amount of information in the output activity, a fact that is reflected by the specific structure of the correlations (rather than their magnitudes) [32]. However, such structures are difficult to pinpoint in detail in measured activity [32]. In experiments, it thus remains difficult to evaluate the influence of network-generated noise on stimulus coding.

In the present study, our goal was to take experimentally observed statistics of the population activity as a starting point, and to interpret the structure of the average responses and noise correlations based on outcomes of model networks. Varying network properties such as the architecture of connections changed both the pattern of average activity and the noise correlations, both of which influence the accuracy of the neural code. As our mathematical results indicate, the effect of recurrent dynamics on information coding depends primarily on the magnitude of the noise generated internally. Our toy model, as well as the investigation presented in Refs. [18, 30], suggests that, generically, recurrent amplification does not improve information coding. The reason is essentially that, in a recurrent network, the noise inherent to the spike generation mechanism is fed back in the network as ‘input noise’, which in turn amplifies the ‘output noise’. This amplification harms the signal-to-noise ratio. By contrast, in a feed-forward network, only the ‘output noise’ in spike affects the code; in Poisson neurons, an increase in this variability is overcompensated by an increase in the firing rate, so that the signal-to-noise ratio improves. Our recurrent network model allows us to estimate the ‘amplification factor’ in an experimental population, as well as the consequent strength of the noise, provided simplifying assumptions such as that of a random effective network architecture.

### Variability in the mouse auditory cortex

We tested the consistency of different scenarios involving interacting Poisson neurons with measured neural population responses, and we interpreted the observed variability in terms of a recurrent network model. The measured population activity was given as spike counts in 250 ms bins, so that correlations were defined over a relatively slow timescale and, therefore, did not take rapid temporal modulation (as noted in, e.g., Ref. [49]) into account. Both experimental variability and correlations were high [50], so that the effects of correlations were potentially strong, and amenable to an analysis such as ours. In general, variability and correlations can be high in anesthetized animals [11], but noise and signal correlations were noted to be somewhat weaker in experiments that are comparable to the one we consider [51]. While it is difficult to rule out that part of the correlations result from experimental artifacts relating to the calcium imaging technique used in the experiments (e.g., scattering of fluorescent light by the neuropil), intracellular recordings of rates were consistent with calcium recordings [35]. A related point concerns the identification of the deconvolved fluorescence signal with the neural response: if the latter is inferred up to a multiplicative factor, the numerical values of the inferred covariances will be affected by the square of this factor. Such a factor, however, would not change the functional form of the relations between firing rates and covariances, but only the numerical values of inferred parameters. Furthermore, calcium imaging operates on a slow time scale as compared to the dynamics of individual spikes. In this work, we were interested in the coarse population activity and variability, and therefore considered spike counts in a fixed window, thereby disregarding the precise temporal dynamics of neural activity. The latter may indeed be relevant, especially in auditory processing [52, 53].

In comparing the data to our model results, we did not to attempt at inferring the strength of individual (effective) connections between neurons; this would require a much larger volume of data. Instead, we focused on the parameters of the distribution of effective, random connections. This approach, in the context of a recurrent network model, allowed us to capture not only the scaling of variances and covariances with firing rates, but also the relation between signal correlation and noise correlation, and the effect of shuffling away covariances on stimulus discrimination. Furthermore, differences among experimental populations of neurons could be attributed to changes in the effective connectivity within populations.

Recent studies [7, 9, 11–14] have proposed the mechanism of common gain fluctuation to explain correlated variability across a population. In this work, we identify similarities and differences of correlated variability that arises in recurrent models vs. feed-forward models such as the common-gain-fluctuation model. Estimated model parameters indicate recurrent amplification and suggests that the portion of the variability that can be traced back to spike generation in the population—noise harmful to coding—is comparable in magnitude to the external input noise. Interpreting correlations in the framework of a random network is obviously not a new idea; our results illustrate the fact that subtle differences in the output statistics can lead to very different interpretations on network architecture.

Our data analysis suffers from an unknown offset due to putative baseline spiking rates, the limited volume of the data, and the low temporal resolution. In this context, we compared different prototypical scenarios, but did not consider mixed models. In experimental populations of neurons, the statistics of activity can obviously result from a combination of feed-forward and recurrent processes. An likely scenario is one in which a feed-forward network provides correlated input to a recurrent network from which recordings are carried out. For simple choices of model stimulus sets, pure network models will not explain all aspects of the data. Examining the relative contributions of recurrent and feed-forward processes may be relevant to understanding the origin and role of correlated variability. As a first step, here, we provide some characterizations of the outcome of each process taken individually.

## Supporting information

S1 Appendix Details of derivations, numerical simulations, fits to data. (PDF)

S2 Appendix Simple two-population model. (PDF)

S1 Fig Effect of covariances on stimulus discrimination, in the models of feed-forward networks with shared input and with common gain fluctuation. (Compare to Fig 4). Parameters for the feed-forward network with shared input are as in Fig 2. As average responses are arbitrary in the gain fluctuation model, identical average responses as in the shared input model were assumed, and the variance of gain fluctuations set to *V*_ext_ = 0.53. A, C: Distributions of cosines of angle between the diagonal and the difference of average responses for stimulus pairs, in feed-forward networks with shared input (A) and common gain fluctuation (C), for different values of the parameter *ρ*. B, D: Discrimination ratios in the cases of correlations generated by shared input (B) and common gain fluctuation (D), for the same set of values of the parameter *ρ*.
(TIF)
