# Supplementary material for: Interpretation of correlated neural variability from models of feed-forward and recurrent circuits
Source: PLoS Comput Biol. 2018 Feb 6;14(2):e1005979. doi: 10.1371/journal.pcbi.1005979 (PMC5833435; doi:10.1371/journal.pcbi.1005979)
Supplement: S1 Appendix — (PDF) [file pcbi.1005979.s001.pdf]

Interpretation of correlated neural variability from models of feed-forward and recurrent circuits

S1 Appendix - Details of derivations, numerical simulations, fits to data

Volker Pernice[1,2], Rava Azeredo da Silveira [1,2,3*]

1 Department of Physics, Ecole Normale Supérieure, 75005 Paris, France
2 Laboratoire de Physique Statistique, Ecole Normale Supérieure, PSL Research University; Université Paris Diderot Sorbonne Paris-Cité; Sorbonne Universités UPMC Univ Paris 06; CNRS, Paris, France
3 Princeton Neuroscience Institute, Princeton University, Princeton, NJ 08544, USA

* rava@ens.fr

1 Numerical simulations: details and parameters

References to equations and figures in the main text are preceded by the letter "M" and references to supplement S2 by "S2" in this supplement.

In Fig. M2, covariances were calculated for recurrent networks from Eq. (M12), with the addition of a rate offset $a = 4$, so that $C = (\mathbb{I} - G)^{-1}\big(D[r] + a + D[V_{\text{ext}}]\big)(\mathbb{I} - G^T)^{-1}$. The stimulus ensemble consisted of 200 input vectors r_{ext} of size $N = 60$, with entries independently chosen from a normal distribution with mean 0.2 and standard deviation 1. Rates r were calculated as in Eq. (M7), and we assumed Poisson input with $V_{\text{ext}} = |r_{\text{ext}}|$. For the networks, four connectivity matrices G of size $N = 60$ were generated. Their entries were chosen from a normal distribution with standard deviation $1.5/N$ and mean $0.8/N - 0.9/N$, respectively. For the analytic predictions from Eqs. (M24)-(M27), the mean and variance of the transfer matrix $B = (\mathbb{I} - G)^{-1}$ were calculated numerically.

In Fig. M2, covariances were calculated for recurrent networks from Eq. (M12). For the networks, four connectivity matrices G of size $N = 60$ were generated. Their entries were chosen from a normal distribution with standard deviation $1.5/N$ and mean $0.8/N - 0.9/N$, respectively. For the input ensemble for the recurrent network, elements of 200 random input vectors r_{ext} of length $N = 60$ were chosen from a normal distribution with mean 0.4 and variance 2. Rates r were calculated as in Eq. (M7), and we assumed Poisson input with $V_{\text{ext}} = |r_{\text{ext}}|$. For rates and covariances of the feed-forward scenario, we used Eqs. (M16) and (M17). We set $F = 10 \cdot B$, and scaled the input vectors by a factor 1/10 to obtain rates and correlations of comparable size as in the recurrent network. Negative output rates were set to zero. For the gain fluctuation model, responses of the network with $\rho = 1.4$ were used as the ensemble of stimulus responses. From these, covariances were calculated, see Eq. (M19), for which the variance of external fluctuations V_{ext} was varied. For this figure, no rate offset was used, $a = 0$. For the analytic predictions from Eqs. (M24)-(M27), the mean and variance of the transfer matrix $B = (\mathbb{I} - G)^{-1}$ were calculated numerically.

The networks in Fig. M3C were identical to the ones used above. 50 stimuli r_{ext} with random entries were generated with mean 0.2 and standard deviation 1.5. Inputs for different neurons were correlated with correlation coefficient $c_{\text{in}} = 0.05$ across stimuli. Rate offset was 4.

In Fig. M4, networks and stimulus parameters were the same as in Fig. M3, but there were no input signal correlations, $c_{\text{in}} = 0$.

In Fig. S2 1, the network connects two populations of 100 neurons each. In a first pair of stimuli (Fig. S2 1B), neurons in the population that was more strongly excited received an input of $1 + \Delta$, with $\Delta = 0.2$, and the other neurons received an input of 1. For a second pair of stimuli (Fig. S2 1F), the two populations received inputs of $1 + \Delta$ and $1 - \Delta$, respectively. Each neuron had a fixed number of n_{EE} postsynaptic partners within the same populations. Coupling strength between connected neurons was set to $g = 0.01$. We chose a population coupling within populations of $\Gamma_s = 0.2$, and the number of connections between neurons within populations such that $\Gamma_s = n_{EE}g$. Connections across populations were chosen accordingly to realize across coupling values $\Gamma_c = 0, \ldots, 0.4$. To visualize the effect of the increase in internally generated noise due to increased rates (panel B), we compared the covariances $\Sigma = B^p D[R](B^p)^T$ for increasing Γ_c to the ones where in $D[R]$ the rates of a network with $\Gamma_c = 0$ were used.

In Fig. S2 1E, we calculated $S_{original}$ by inserting the population covariances and responses in Eq. (M22) for the two stimuli, in the correlated case. For the shuffled case, the full matrix covariance matrix C was generated and the off-diagonal elements set to 0, such that

$$\Sigma_{shuffled} = \begin{pmatrix} \sum_{i \in E} C_{ii} & 0 \\ 0 & \sum_{i \in E'} C_{ii} \end{pmatrix}. \tag{1}$$

In Fig. S2 1F, $\Gamma_c = 0.4$ and $\Gamma_s = 0.2$. In Fig. S2 1G, we fixed, in the population transfer matrix, P, the ratio $\rho = \text{var}(P)/\langle P \rangle^2$, to the value $\rho = 1$. Then, $\langle P \rangle$ was varied between 0.1 and 5, with $\text{var}(P)$ fixed. From $\langle P \rangle$ and $\text{var}(P)$, we calculated P_s and P_c (see Supplement S2).

2 Common framework for the analysis of noise correlations

Here we formulate the three model scenarios described in Methods as special cases of the interacting point processes framework defined in Eq. (M6). The derivation is similar as in [1, 2]. We consider an extended network of two populations which receive only constant input $r_{\text{full}}(t) \equiv r_{\text{full}}$. Neurons are divided into an observed population O and an unobserved external population U. The external population projects to the observed population, but does not receive feedback. The coupling matrix of integrated kernels of the full system is a block matrix of the shape

$$G_{\text{full}} = \begin{pmatrix} E & 0 \\ F & G \end{pmatrix}. \tag{2}$$

The coupling between nodes within the external network is described by the matrix E. If $E = 0$, external inputs are independent Poisson processes. The feed-forward weights to the observed network are defined by F, and by G the recurrent connections within the observed population. The input vector to the system is $r_{\text{full}} = (r_0, 0)$. The components of the vector r_0 together with E determine the firing rates of the input nodes. The 0 represents a vector of zeros, so that there is no constant input directly to the observed population.

The time dependent firing rates of neurons $k \in U$ in the external input population are determined by

$$\tilde{r}_k(t) = \sum_{l \in U} \int_0^\infty \tilde{e}_{kl}(\tau)\tilde{s}_l(t - \tau)d\tau + r_{0,k} \tag{3}$$

and the rates of neurons $i \in O$ in the observed population by

$$\tilde{r}_i(t) = \sum_{j \in O} \int_0^\infty \tilde{g}_{ij}(\tau)\tilde{s}_j(t - \tau)d\tau + \sum_{k \in U} \int_0^\infty \tilde{f}_{ik}(\tau)\tilde{s}_k(t - \tau)d\tau. \tag{4}$$

The transfer matrix of the full system is

$$B_{\text{full}} = (\mathbb{I} - G_{\text{full}})^{-1} = \begin{pmatrix} (\mathbb{I}-E)^{-1} & 0 \\ (\mathbb{I}-G)^{-1}F(\mathbb{I}-E)^{-1} & (\mathbb{I}-G)^{-1} \end{pmatrix} \equiv \begin{pmatrix} B_E & 0 \\ BFB_E & B. \end{pmatrix} \quad (5)$$

The average rates of the system are, from applying Eq. (M7) to the full system,

$$(\mathbb{I} - G_{\text{full}})^{-1} r_{\text{full}} = B_{\text{full}} r_{\text{full}} = (B_E r_0, BFB_E r_0)^T \equiv (r_{\text{ext}}, r)^T, \quad (6)$$

where $r_{\text{ext}} = B_E r_0$ are the rates of the input neurons and $r = BFB_E r_0 = BFr_{\text{ext}}$ correspondingly, the rates of the observed neurons. From Eq. (M12), the covariance matrix is given by

$$C_{\text{full}} = \begin{pmatrix} B_E D[r_{\text{ext}}] B_E^T & B_E D[r_{\text{ext}}] B_E^T F^T B^T \\ BFB_E D[r_{\text{ext}}] B_E^T & B\Big(D[r] + FB_E D[r_{\text{ext}}] B_E^T F^T\Big) B^T \end{pmatrix}. \quad (7)$$

The block $C_E \equiv B_E D[r_{\text{ext}}] B_E^T$ at the upper left describes the covariances of the external population. The block at the lower right,

$$C \equiv B(D[r] + FC_E F^T) B^T \quad (8)$$

describes how covariances in the observed network depend on the properties of the unobserved input neurons.

The recurrent network scenario is obtained for an input population and an output population of equal size N, with $F = \mathbb{I}$ and any diagonal E. In this case, C_E is diagonal, and we can identify the elements on the diagonal of C_E with V_{ext}. For the feed-forward scenario set $G = 0$ and again identify the diagonal elements of C_E with V_{ext}. The gain fluctuation model is obtained for $G = 0$, and if the input population consists of a single neuron. The matrix F then corresponds to a single column vector, and $C = D[Fr_{\text{ext}}] + FC_E F^T$. With $r = Fr_{\text{ext}}$ and setting $V_{\text{ext}} = C_E / r_{\text{ext}}^2$ (r_{ext} is a number in this case), $C = D[r] + rr^T V_{\text{ext}}$.

3 Population response statistics in the three network models

3.1 Recurrent network model

The response distributions in the recurrent network are characterized by the average and covariances of responses given in Eqs. (M7) and (M12),

$$r = Br_{\text{ext}} \quad (9)$$

and

$$C = BD[r_{\text{eff}}]B, \quad (10)$$

with $r_{\text{eff}} = r + a + V_{\text{ext}}$, including an offset a. We compare this model to the alternative scenarios described in the following sections with respect to the relation between average (co)variances and the projected variances and r, as well as signal and noise correlations.

We first derive Eqs. (M23) and (M24). The average covariance is

$$\langle C_{ij} \rangle_{i \neq j} = \frac{1}{N(N-1)} \sum_{i \neq j, k} B_{ik} B_{jk} r_{\text{eff},k} \approx N \langle B_{ik} B_{jk} \rangle_{ijk} \langle r_{\text{eff},k} \rangle_k. \quad (11)$$

We assume that N is sufficiently large and that the external input characterized by $V_{\text{ext}}, r_{\text{ext}}$ does not depend on the recurrent network. In that case $r_{\text{eff},k}$ is approximately uncorrelated to B_{ik} (including the contribution of r_k, because $\langle B_{ij} r_k \rangle = \langle B_{ij} \sum_l B_{kl} r_{\text{ext},l} \rangle \approx$

$\langle B_{ij}\rangle\langle\sum_l B_{kl}r_{\text{ext},l}\rangle$). The term $\langle r_{\text{eff}}\rangle = \langle r\rangle + a + \langle V_{\text{ext}}\rangle$ is linear in $\langle r\rangle$, with an uncorrelated contribution $\langle V_{\text{ext}}\rangle$, see the following section. If the elements of B are pairwise independent,

$$\langle C_{ij}\rangle_{i\neq j} = N\langle B\rangle^2(\langle r\rangle + a + \langle V_{\text{ext}}\rangle). \tag{12}$$

Under the same assumptions, the sum of variances is

$$\begin{aligned}\sigma^2_{all} &= \sum_{i,k} B^2_{ik} r_{\text{eff},k} = \sum_{ik} B^2_{ik}(V_{\text{ext},k} + r_k + a)\\ &\approx N\langle B^2\rangle \sum_k (V_{\text{ext},k} + r_k + a) = N^2\langle B^2\rangle(\langle r\rangle + a + \langle V_{\text{ext}}\rangle).\end{aligned} \tag{13}$$

Again, the term $N^2\langle B^2\rangle\langle V_{\text{ext}}\rangle$ only contributes with an offset to the relation between $\langle r\rangle$ and $\langle C_{ii}\rangle_i = \sigma_{all}/N$.

Next, we show that the projected variances in this scenario have a similar dependence on $\langle r\rangle$ as the ones in the feed-forward model. The projected variance on the diagonal is

$$\sigma^2_d = \frac{1}{N}\sum_{ij,k} B_{ik}B_{jk} r_{\text{eff},k} \approx N^2\langle B\rangle^2(\langle r\rangle + a + \langle V_{\text{ext}}\rangle). \tag{14}$$

Consequently, in the ratio $\sigma^2_d/\sigma^2_{all}$ the dependence on $\langle r\rangle$ is canceled out.

The projection along the mean response direction,

$$\sigma^2_\mu = \sum_{ij,k} B_{ik}B_{jk}\Big(r_k + a + V_{\text{ext},k}\Big)\bar{r}_i\bar{r}_j \approx N^3\frac{\langle r\rangle^2}{|r|^2}\langle B\rangle^2(\langle r\rangle + a + \langle V_{\text{ext}}\rangle), \tag{15}$$

depends strongly on $\langle r\rangle$. Because $\cos(d,r) = 0$ implies $\langle r\rangle = 0$, $\sigma^2_\mu/\sigma^2_{all} = 0$ in this case, and for $\cos(d,r) = 1$, with $|r|^2 = N\langle r\rangle^2$ one gets $\sigma^2_\mu/\sigma^2_{all} = \langle B\rangle^2/\langle B^2\rangle$.

Apart from the raw covariances, we are interested in the average noise correlation coefficient (across stimuli and neuron pairs),

$$c_N = \langle\frac{C_{ij}(s)}{\sqrt{C_{ii}(s)C_{jj}(s)}}\rangle_{s,i\neq j}. \tag{16}$$

For a given stimulus, we assume that the C_{ij} are approximately independent, so that we can write

$$\langle\frac{C_{ij}(s)}{\sqrt{C_{ii}(s)C_{jj}(s)}}\rangle_{i\neq j} \approx \frac{\langle C_{ij}(s)\rangle_{i\neq j}}{\langle C_{ii}(s)\rangle_i}. \tag{17}$$

From Eqs. (12) and (13),

$$c_N = \frac{N\langle B\rangle^2(\langle r\rangle + a + \langle V_{\text{ext}}\rangle)}{N\langle B^2\rangle(\langle r\rangle + a + \langle V_{\text{ext}}\rangle)} = \frac{\langle B\rangle^2}{\langle B^2\rangle}, \tag{18}$$

and because $\langle B^2\rangle = \text{var}(B) + \langle B\rangle^2$ we can rewrite c_N as

$$c_N = \frac{1}{1 + \text{var}(B)/\langle B\rangle^2} = \frac{1}{1+\rho}. \tag{19}$$

We see that the correlation coefficient depends on the relative variability of the network elements. The variances, resulting from the variances of the input channels, are determined by the mean of the square elements of the network. By contrast, the covariances depend on the effective weights of inputs to the neuron pairs, and hence on the square of the mean weight. Correspondingly, we consider the signal covariances $C^S_{ij}(s) = \text{cov}(r_i(s), r_j(s))_s$,

$$C^S_{ij} = \text{cov}(\sum_{kl} B_{ik}r_{\text{ext},k}(s), B_{jl}r_{\text{ext},l}(s))_s = \sum_{kl}\text{cov}(B_{ik}r_{\text{ext},k}(s), B_{jl}r_{\text{ext},l}(s))_s. \tag{20}$$

For $i \neq j$, one gets

$$\begin{aligned} C_{ij}^S &= \sum_{kl} B_{ik} B_{jk} \mathrm{cov}(r_{\mathrm{ext},k}(s), r_{\mathrm{ext},l}(s))_s \\ &= \sum_k B_{ik} B_{jk} \mathrm{var}(r_{\mathrm{ext},k}(s))_s + \sum_{k \neq l} B_{ik} B_{jk} \mathrm{cov}(r_{\mathrm{ext},k}(s), r_{\mathrm{ext},l}(s))_s. \end{aligned} \tag{21}$$

Averaged across neurons

$$\langle C_{ij}^S \rangle_{i \neq j} = N \langle B \rangle^2 \mathrm{var}(r_{\mathrm{ext}}) + N(N-1) \langle B \rangle^2 c_{\mathrm{in}} \mathrm{var}(r_{\mathrm{ext}}). \tag{22}$$

For the signal variances, $i = j$, which correspond to the variance of the rates across stimuli,

$$\begin{aligned} \langle C_{ii}^S(s) \rangle_s &= \sum_k \mathrm{var}(B_{ik} r_{\mathrm{ext},k}) + \sum_{k \neq l} \mathrm{cov}(B_{ik} r_{\mathrm{ext},k}, B_{il} r_{\mathrm{ext},l}) \\ &= \sum_k B_{ik}^2 \mathrm{var}(r_{\mathrm{ext},k}) + \sum_{k \neq l} \mathrm{cov}(r_{\mathrm{ext},k}, r_{\mathrm{ext},l}) B_{ik} B_{il}. \end{aligned} \tag{23}$$

Averaged across neurons, with $\langle B^2 \rangle = \langle B \rangle^2 + \mathrm{var}(B)$:

$$\langle C_{ii}^S(s) \rangle_{s,i} = N \mathrm{var}(r_{\mathrm{ext}})(\langle B \rangle^2 + \mathrm{var}(B)) + N(N-1) \langle B \rangle^2 \mathrm{var}(r_{\mathrm{ext}}) c_{\mathrm{in}}. \tag{24}$$

Their ratio is

$$\begin{aligned} \frac{\langle C_{ij} \rangle}{\langle C_{ii} \rangle} &= \frac{N \langle B \rangle^2 \mathrm{var}(r_{\mathrm{ext}})(1 + (N-1) c_{\mathrm{in}})}{N \mathrm{var}(r_{\mathrm{ext}})(\langle B \rangle^2 + \mathrm{var}(B)) + N(N-1) \langle B \rangle^2 \mathrm{var}(r_{\mathrm{ext}}) c_{\mathrm{in}}} \\ &= \frac{1 + (N-1) c_{\mathrm{in}}}{1 + \mathrm{var}(B)/\langle B \rangle^2 + (N-1) c_{\mathrm{in}}}. \end{aligned} \tag{25}$$

This results in an approximate expression for the average signal correlation coefficient,

$$c_S = \langle \frac{C_{ij}^S}{\sqrt{C_{ii}^S C_{jj}^S}} \rangle_{i,j} \approx \frac{\langle C_{ij}^S \rangle}{\langle C_{ii}^S \rangle} = \frac{1 + (N-1) c_{\mathrm{in}}}{1 + \mathrm{var}(B)/\langle B \rangle^2 + (N-1) c_{\mathrm{in}}}. \tag{26}$$

3.2 Feed-forward network model with shared inputs

Here we derive characteristic relations for the response statistics resulting in a feed-forward network. They illustrate qualitative differences between the predictions of different models and will be used to extract model parameters from the data. In the feed-forward model the mean responses and covariances are, from Eqs. (M16) and (M17) and allowing an offset in observed rates,

$$r = F r_{\mathrm{ext}}, \tag{27}$$

$$C_{ij} = \delta_{ij}(r_i + a) + \sum_k F_{ik} F_{jk} V_{\mathrm{ext},k}. \tag{28}$$

In contrast to the recurrent network model (see above), the average covariance is not correlated to the population averaged rate $\langle r \rangle$ across stimuli, Eq. (M26). This follows after taking the average across neurons:

$$\langle C_{ij} \rangle_{i \neq j} = \frac{1}{N(N-1)} \sum_{i \neq j, k} F_{ik} F_{jk} V_{\mathrm{ext},k} \approx N \langle F_{ik} \rangle_{ik} \langle F_{jk} \rangle_{jk} \langle V_{\mathrm{ext},k} \rangle_k = N \langle F \rangle^2 \langle V_{\mathrm{ext}} \rangle. \tag{29}$$

Input variances V_{ext} are assumed to be independent of the network structure and F_{ik} independent of F_{jk}, which means that the strengths of connection of an external neuron to different internal neurons are independent.

If $\langle C_{ij}\rangle_{i\neq j}$ is to be uncorrelated to $\langle r\rangle$ across stimuli, the average input variance $\langle V_{\rm ext}\rangle$ needs to be approximately uncorrelated to $\langle r\rangle$. This holds in our model even though $r_k = \sum_i F_{ik} r_{{\rm ext},k}$ and in each *input* channel the variance equals the strength of the input, $V_{{\rm ext},k} = |r_{{\rm ext},k}|$, if either the distribution of inputs across neurons is approximately symmetric around 0, or the distribution of column sums $\sum_i F_{ik}$ of the feed-forward matrix (across columns) is symmetric around 0. The first case can be realized in a feed-forward network with a similar number of excitatory and inhibitory input channels. Intuitively, inhibitory inputs decrease the average output, but contribute positively to the average variance, and thus decorrelate the two quantities.

Formally, one compares the random variable $\sum_k r_k = \sum_{ik} F_{ik} r_{{\rm ext},k} = \sum_k r_{{\rm ext},k} \sum_i F_{ik}$ to the variable $\sum_k V_{{\rm ext},k} = \sum_k |r_{{\rm ext},k}|$. A random variable x is uncorrelated to its absolute value $|x|$ if its distribution is symmetric around 0. Here, the relevant variables are the elements of the input vector $r_{\rm ext}$, and their distribution is approximately symmetric around 0, if the variance ${\rm var}(r_{\rm ext})$ is much larger than their squared mean, $\langle r_{\rm ext}\rangle$, that is if $\rho_{\rm ext} = {\rm var}(r_{\rm ext})/\langle r_{\rm ext}\rangle^2 \gg 1$. Consequently, if the variance of external inputs across stimuli is high, the population averaged covariances in a feed-forward network are uncorrelated to the population response.

In the following, we motivate that the stimulus dependence of the variances projected along different directions in the feed-forward model is different from the one in the gain fluctuation model. The sum of the variances is

$$\sigma^2_{all} = N(\langle r\rangle + a) + \sum_{i,k} F^2_{ik} V_{{\rm ext},k} \approx N\big(\langle r\rangle + a\big) + N^2 \langle V_{\rm ext}\rangle\langle F^2\rangle. \tag{30}$$

Due to the Poisson spike generation, there is a linear contribution in $\langle r\rangle$ to the average variance, $\langle C_{ii}\rangle_i = \sigma^2_{all}/N$. This variability does not contribute to covariances, because spikes are generated independently across neurons, in contrast to the recurrent model, where the spiking or not spiking of a neuron directly influences post-synaptic firing rates.

The variance projected onto the diagonal direction is

$$\sigma^2_d = \langle r\rangle + a + \frac{1}{N}\sum_{ijk} F_{ik}F_{jk}V_{{\rm ext},k} \approx \langle r\rangle + a + N^2\langle V_{\rm ext}\rangle\langle F\rangle^2. \tag{31}$$

Consequently, the ratio $\sigma^2_d/\sigma^2_{all}$ depends only weakly on $\langle r\rangle$ if the term $N\langle V_{\rm ext}\rangle\langle F\rangle^2$ is large against $\langle r\rangle + a$, that is if the sum of covariances is larger than the sum of variances. This is the case, if correlation coefficients are larger than of the order $O(1/N)$. The variance projected onto the direction of the mean response is

$$\sigma^2_\mu = \sum_i (r_i + a)\bar r_i^2 + \sum_{ijk} F_{ik}F_{jk}V_{{\rm ext},k}\bar r_i \bar r_j \approx a + \frac{\sum_i r_i^3}{|r|^2} + \frac{\langle r\rangle^2}{|r|^2} N^3 \langle F\rangle^2 \langle V_{\rm ext}\rangle. \tag{32}$$

To see that the ratio $\sigma^2_\mu/\sigma^2_{all}$ strongly depends on the population response, in contrast to $\sigma^2_d/\sigma^2_{all}$, assume again that the sum across variances in the first term $a + \frac{\sum_i r_i^3}{|r|^2}$ is not too large against the sum across covariances in the second term. The second term depends strongly on $\langle r\rangle$: it is 0 for $\cos(r,d) = 0$. For $\cos(r,d) = 1$ it becomes $N^2\langle F\rangle^2\langle V_{\rm ext}\rangle$. In this case, $\sigma^2_\mu/\sigma^2_{all}$ is not too small, if $(\langle r\rangle + a)/(NV_{\rm ext})$ is not much larger than 1 (assuming that $\langle F^2\rangle/\langle F\rangle^2$ is of order one).

As for the recurrent network, we approximate c_N as

$$c_N \approx \frac{\langle C_{ij}\rangle_{s,i\neq j}}{\langle C_{ii}(s)\rangle_{s,i}}. \tag{33}$$

Then, from Eqs. (29) and (30),

$$c_N = \frac{N\langle F\rangle^2\langle V_{\rm ext}\rangle}{\langle r\rangle + a + N\langle V_{\rm ext}\rangle\langle F^2\rangle}. \tag{34}$$

Signal correlations can be calculated analogously as for the recurrent model.

3.3 Feed-forward network model with common gain fluctuation

We derive relations between covariances and average response in the gain fluctuation model, in particular the scaling of average covariances with average rates and the orientation of the response distribution measured by the projections of the variances in different directions. From Eq. (M19), pairwise covariances are directly related to average responses,

$$C_{ij} = \delta_{ij} r_i + r_i r_j V_{\text{ext}}. \tag{35}$$

The relation between average covariance and the population averaged response, Eq. (M27), follows from

$$\langle C_{ij} \rangle_{i \neq j} = \frac{1}{N(N-1)} \sum_{i \neq j} r_i r_j V_{\text{ext}} \approx \frac{V_{\text{ext}}}{N^2} \Big(\sum_i r_i \Big) \Big(\sum_j r_j \Big) = V_{\text{ext}} \langle r \rangle^2, \tag{36}$$

where the terms r_i^2 can be neglected if N is large.

To measure changes of the response distribution across stimuli, we use the variance projected in the direction of mean response and diagonal direction, σ_μ^2 and σ_d^2, respectively. In the following, we give an argument that in this model σ_μ^2 does not strongly depend on the stimulus, while σ_d^2 does.

Both quantities are normalized by the sum of the variances

$$\sigma_{all}^2 = \sum_i C_{ii} = \sum_i (r_i^2 V_{\text{ext}} + r_i) = |r|^2 V_{\text{ext}} + N \langle r \rangle. \tag{37}$$

The variance projected in the diagonal direction $\bar{d} = (1, \ldots, 1)^T / \sqrt{N}$ is

$$\sigma_d^2 = \sum_{ij} C_{ij}(s) \bar{d}_i \bar{d}_j = \sum_{ij} \Big(r_i r_j V_{\text{ext}} + \delta_{ij} r_i \Big) / N = N V_{\text{ext}} \langle r \rangle^2 + \langle r \rangle. \tag{38}$$

To see that there is a strong dependence of $\sigma_d^2 / \sigma_{all}^2$ on stimulus direction, note that

$$\cos(d, r) = \bar{d} \bar{r}^T = \sum_{i=1}^{N} \frac{1}{\sqrt{N}} \frac{r_i}{|r|} = \sqrt{N} \langle r \rangle / |r|. \tag{39}$$

For non-vanishing r, it follows from $\cos(d, r) = 0$ that $\langle r \rangle = 0$ and in this case $\sigma_d^2 / \sigma_{all}^2 = 0$.

If $\cos(d, r) = 1$, $r \propto d$ and $|r|^2 = N \langle r \rangle^2$, and so $\sigma_d^2 / \sigma_{all}^2 = \frac{N V_{\text{ext}} \langle r \rangle^2 + \langle r \rangle}{N V_{\text{ext}} \langle r \rangle^2 + N \langle r \rangle}$. This value is of the order of one, if the ratio of $V_{\text{ext}} \langle r \rangle^2$ and $\langle r \rangle$, which is of the order of the average noise correlation coefficient, is not very small (in comparison to one). Consequently, if noise correlations are not too small, the normalized variance projected on the diagonal direction strongly depends on the direction of the response vector.

The variance projected on the mean direction is

$$\sigma_\mu^2 = \sum_{ij} \bar{r}_i \bar{r}_j C_{ij} = \sum_{ij} \bar{r}_i \bar{r}_j (r_i r_j V_{\text{ext}} + \delta_{ij} r_i) > V_{\text{ext}} \sum_{ij} r_i^2 r_j^2 / |r|^2 = V_{\text{ext}} |r|^2. \tag{40}$$

Hence, $\sigma_\mu^2 / \sigma_{all}^2 > \frac{V_{\text{ext}} |r|^2}{|r|^2 V_{\text{ext}} + N \langle r \rangle}$, which depends only weakly on $\langle r \rangle$, if $N \langle r \rangle$ is not much bigger than $V_{\text{ext}} |r|^2 \geq V_{\text{ext}} N \langle r \rangle^2$, that is, once again, if noise correlations are not too small.

4 Comparisons between data and models

4.1 Estimating network model parameters

We extract parameters of the recurrent and feed-forward network models from the data, both to test the consistency of the models with the data and to interpret the observed variability.

Because rates and covariance matrices were measured for many different stimuli and thus provide a large number of constraints, one approach would be to infer as much information as possible about the full connectivity matrices B or F from the data. However, due to the relatively small number of trials for each stimulus, we use a model with few parameters. The set of parameters consists of the network parameters $\langle B \rangle$ and $\mathrm{var}(B)$ ($\langle F \rangle$ and $\mathrm{var}(F)$, respectively) as well as the parameters of the input ensemble, $\langle r_{\mathrm{ext}} \rangle$, $\mathrm{var}(r_{\mathrm{ext}})$ and c_{in}.

In particular, we want to infer the ratios $\rho_{\mathrm{ext}} = \mathrm{var}(r_{\mathrm{ext}})/\langle r_{\mathrm{ext}} \rangle^2$ and $\rho = \mathrm{var}(B)/\langle B \rangle^2$ (and correspondingly for F). Based on these, we can generate surrogate data to test if the observed scaling of average covariances with average rates is more consistent with the recurrent or the feed-forward model, Eq. (M24) or (M26). The experimental data provides constraints in the form of the population and stimulus averaged rates, their variances and the noise and signal correlation coefficients. In the models, rates are given by Eqs. (M7) and (M16), respectively. The population averaged mean response thus is

$$\langle r \rangle = N \langle B \rangle \langle r_{\mathrm{ext}} \rangle \text{ or } \langle r \rangle = N \langle F \rangle \langle r_{\mathrm{ext}} \rangle. \tag{41}$$

The variance of rates across stimuli, see Eq. (24), is

$$\mathrm{var}(r) = N \mathrm{var}(r_{\mathrm{ext}}) (\langle B \rangle^2 + \mathrm{var}(B) + (N-1) \langle B \rangle^2 c_{\mathrm{in}}) \tag{42}$$

such that the relative variance of rates

$$\frac{\mathrm{var}(r)}{\langle r \rangle^2} = \frac{\mathrm{var}(r_{\mathrm{ext}})}{\langle r_{\mathrm{ext}} \rangle^2} \frac{1 + \rho + (N-1) c_{\mathrm{in}}}{N} \tag{43}$$

depends on the input signal to noise ratio $\rho_{\mathrm{ext}} = \frac{\mathrm{var}(r_{\mathrm{ext}})}{\langle r_{\mathrm{ext}} \rangle^2}$ and the variability of the network elements, $\rho = \frac{\mathrm{var}(B)}{\langle B \rangle^2}$, or $\rho = \frac{\mathrm{var}(F)}{\langle F \rangle^2}$ respectively.

The estimates for the remaining parameters ρ and c_{in} are obtained from the measured values of the ratio of covariances to variances, which correspond approximately to the average coefficients of noise and signal correlations,

$$\langle \frac{\langle C_{ij} \rangle_{i \neq j}}{\langle C_{ii} \rangle_i} \rangle_s \approx c_N, \tag{44}$$

and

$$\frac{\langle \mathrm{cov}(r_i, r_j) \rangle_{i \neq j}}{\langle \mathrm{var}(r_i) \rangle_i} \approx c_S, \tag{45}$$

using Eqs. (19) and (26). Together with Eq. (43), these relations provide the necessary constraints for the network models. Strictly speaking, these equations are valid only for the recurrent network, while for the feed-forward model, Eq. (34) is relevant. In this case ρ is overestimated, which results in a lower bound for ρ_{ext}, and thus a conservative estimate of the input variability.

Under additional assumptions, we can also choose the absolute values of the parameters, for example $\langle r_{\mathrm{ext}} \rangle$ and $\langle F \rangle$, such that mean rates and mean covariances correspond to experimental ones. The mean rates (41) constrain the product of the two parameters

$$\langle r \rangle = N \langle F \rangle \langle r_{\mathrm{ext}} \rangle. \tag{46}$$

From Eq. (29) it follows that

$$\langle C_{ij} \rangle_{i \neq j} = N \langle F \rangle^2 \langle V_{\mathrm{ext}} \rangle, \tag{47}$$

and we assume that $V_{\mathrm{ext}} = |r_{\mathrm{ext}}|$ to relate mean input and input variance. The distribution of V_{ext} thus is a folded normal distribution, with

$$\langle |r_{\mathrm{ext}}| \rangle = \sqrt{\mathrm{var}(r_{\mathrm{ext}})} \sqrt{2/\pi} e^{-1/2\rho_x} + \langle r_{\mathrm{ext}} \rangle \Big(1 - 2\Phi(-\sqrt{1/\rho_x}) \Big) \tag{48}$$

(with the cumulative normal Φ), and from this one finds

$$\langle C_{ij}\rangle = \langle F\rangle\langle r\rangle\Big[\sqrt{\rho_x}e^{-1/2\rho_x} + 1 - 2\Phi(-\sqrt{1/\rho_x})\Big]. \tag{49}$$

To set the absolute values of input versus network strength we made assumptions regarding the relation between input strength and input variance across trials. If we measure the strength of the dependence between mean response and mean covariance, Eq. (29) by the ratio intercept/slope of a linear fit, however, the result is independent of the absolute values of $\langle F\rangle$ and $\langle r_{\text{ext}}\rangle$, as well as a potential linear factor relating V_{ext} and $|r_{\text{ext}}|$.

4.2 Taking a putative baseline firing rate into account in data fits

If rates are only measured up to an unknown offset a, the variance $\sigma_\mu^2/\sigma_{all}^2$, which is obtained from a projection on the apparent average response $r - a$ may not be constant across stimuli. In this case, the variation of this quantity across stimuli is not a reliable indicator to exclude a gain fluctuation model as the source of observed correlations. However, if a large part of the covariances can be explained by a single component, it should be possible to reconstruct the common origin from the stimulus dependent covariance matrices. From

$$C(s) = \big(r(s) + a\big)^T\big(r(s) + a\big)V_{\text{ext}} + D\big(r(s) + a\big) \tag{50}$$

the vectors $v(s) = r(s) + a$ can be obtained approximately by finding the eigenvector with the largest eigenvalue of C, by neglecting the contribution of $D\big(r(s) + a\big)$. This approximation can also be avoided by applying a factor analysis with a single latent component. Because a is constant across stimuli, up to measurement errors of the estimated covariances, the straight lines through the mean responses $r + x\bar{v}$, for $x \in \mathbb{R}$ and normalized directions $\bar{v}$, will intersect in the point $-a$. The best intersection point of multiple lines in the least square sense is given by

$$-\hat{a} = \Big(\sum_s \mathbb{I} - \bar{v}(s)\bar{v}(s)^T\Big)^{-1}\Big(\sum_s (\mathbb{I} - \bar{v}(s)\bar{v}(s)^T)r(s)\Big) \tag{51}$$

and can be used to correct the average responses to $r'(s) = r(s) + \hat{a}$. We found that the analysis of σ_μ^2 and σ_d^2 based on the corrected responses lead to no qualitative change in the stimulus dependence, indicating that a potential shared component is too weak to be identified based on the available data.

References

1. Hawkes AG. Point spectra of some mutually exciting point processes. J R Stat Soc B. 1971;33(3):438–443.

2. Beck J, Bejjanki VR, Pouget A. Insights from a simple expression for linear fisher information in a recurrently connected population of spiking neurons. Neural Comput. 2011;23(6):1484–1502. doi:10.1162/NECO_a_00125.
