# Supplementary material for: Interpretation of correlated neural variability from models of feed-forward and recurrent circuits
Source: PLoS Comput Biol. 2018 Feb 6;14(2):e1005979. doi: 10.1371/journal.pcbi.1005979 (PMC5833435; doi:10.1371/journal.pcbi.1005979)
Supplement: S2 Appendix — (PDF) [file pcbi.1005979.s002.pdf]

Interpretation of correlated neural variability from models of feed-forward and recurrent circuits

S2 Appendix - Simple two-population model

Volker Pernice[1,2], Rava Azeredo da Silveira [1,2,3*]

1 Department of Physics, Ecole Normale Supérieure, 75005 Paris, France
2 Laboratoire de Physique Statistique, Ecole Normale Supérieure, PSL Research University; Université Paris Diderot Sorbonne Paris-Cité; Sorbonne Universités UPMC Univ Paris 06; CNRS, Paris, France
3 Princeton Neuroscience Institute, Princeton University, Princeton, NJ 08544, USA

* rava@ens.fr

1 Definitions and statement of the 'macroscopic equations' for population firing rates and covariances

References to equations and figures in the main text are preceded by the letter "M".

In order to develop an intuitive understanding of the way in which correlations generated in a recurrent network influence coding, we examine a highly simplified model. The recurrent network consists of two excitatory sub-populations, labeled E and E', each made up of N neurons, see sketch in Fig. 1 A. We ask to what extent their activity discriminates two stimuli when these elicit preferential responses in the two sub-populations, respectively.

The activity in the network is determined by Eqs. (M7) and (M12). For the sake of simplicity, we can set the external variance to zero, such that the input to the network is entirely defined by its mean input, r_{ext}. We assume some further simplifications, for the sake of calculational ease: each neuron in sub-population L projects to a fixed number, n_{KL}, of neurons in sub-population K; all non-zero coupling weights are identical, $G_{ij} = g_E$. Additionally, all neurons in the same population receive identical external input. Then, the two-component vectors $R_{\text{ext}}(s_1) = (1 + \Delta, 1)^T$ and $R_{\text{ext}}(s_2) = (1, 1 + \Delta)^T$ define a pair of stimuli. Each component denotes the input to each neuron in the corresponding population.

We reduce the dimensionality of the system by considering the population activity at a macroscopic level. The average response of population K across trials is defined as

$$R_K = \sum_{k \in K} r_k, \tag{1}$$

and its trial-to-trial variability is described by the population covariance matrix, with elements defined as

$$\Sigma_{KL} = \sum_{k \in K, l \in L} C_{kl}. \tag{2}$$

It turns out that these macroscopic quantities depend only on the overall number of connections between populations, not on a specific network realization. For example, the sum of the rates of neurons in one population depends only on the sum of the inputs to all neurons, but not on how these inputs are distributed among the neurons. This is a consequence of

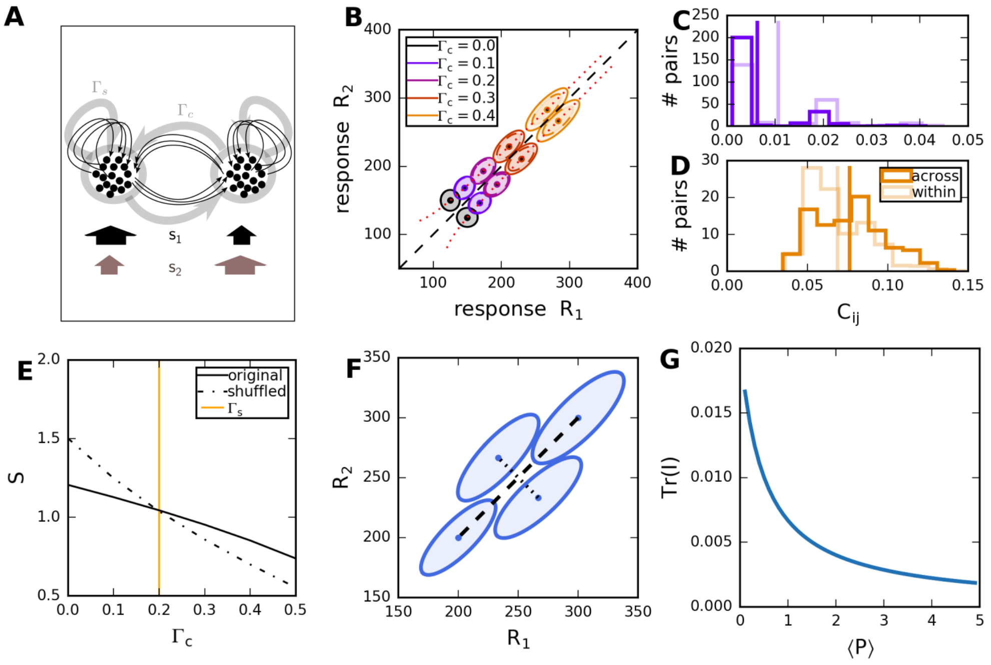

Fig 1. Model of two coupled excitatory populations. A: Illustration of the network architecture. Neurons are coupled with excitatory connections within and across two populations. For each of two stimuli, one population receives stronger input. The network can be described by macroscopic variables. B: Average responses to two stimuli (colored dots on dotted red line) are amplified for stronger cross-coupling (brighter colors); response fluctuations become more correlated. If internally generated noise does not increase with rate, variability is smaller (dashed ellipses). C and D: Distribution of pairwise covariances within (dark bars) and across populations (light bars); vertical lines indicate average. If cross-coupling is weak (C), average covariance is larger within populations than across; if it is strong (D), covariances between neurons of different populations are larger. E: Dependence of stimulus discriminability, S, on Γ_c. Strong coupling implies smaller discriminability and even more so for shuffled trials. F: Ellipses: Response distributions for four stimuli in the recurrent network. Dotted/dashed lines: dimension of stimulus changes for which correlations have positive/negative influence. G: Trace of linear Fisher information matrix as a combined measure of coding of high-dimensional stimulus. Information decreases with average of population coupling. See supplement S1 for numerical values of parameters.

the linearity of the dynamics and the assumption that each neuron has a fixed number of output connections [1, 2], as we show in detail in Sec. 2.

More precisely, we define the coupling within each population, $\Gamma_s = n_{EE}g_E = n_{E'E'}g_E$, and across populations, $\Gamma_c = n_{EE'}g_E = n_{E'E}g_E$, which make up a population coupling matrix

$$\Gamma = \begin{pmatrix} \Gamma_s & \Gamma_c \\ \Gamma_c & \Gamma_s \end{pmatrix}. \tag{3}$$

Applying the definition in Eqs. (1) and (2), we can rewrite the microscopic Eqs. (M7) and (M12) as the population equations

$$R(s) = (\mathbb{I} - \Gamma)^{-1} N R_{\text{ext}}(s) \tag{4}$$

and

$$\Sigma(s) = (\mathbb{I} - \Gamma)^{-1} D[R(s)] (\mathbb{I} - \Gamma)^{-1}. \tag{5}$$

Up to a factor N, these equations are equivalent to the microscopic equations in the restricted context of the two-population model.

2 Derivation of the macroscopic equations

Here, we derive the macroscopic Eqs. (4) and (5). The homogeneity of each of the two sub-populations implies that, for neuron l in population L, the sum $\sum_{k\in K} G_{kl}$ is independent of l, so that we can define the population coupling matrix, Γ, through its elements,

$$\Gamma_{KL} \equiv \sum_{k\in K} G_{kl} = \frac{1}{N}\sum_{k\in K, l\in L} G_{kl} = n_{KL} g_{KL}, \tag{6}$$

where g_{KL} is the weight of a microscopic connection between a neuron in population K and a neuron in population L. We would like to to express R and Σ in terms of Γ. At the microscopic level, the rates, r, and the covariances, C, depend on the transfer matrix, $B = (I - G)^{-1}$. We define an analogous, population transfer matrix, P, that depends only on Γ, and we show that R and Σ can be written in terms of P.

Let

$$P \equiv (\mathbb{I} - \Gamma)^{-1} = \mathbb{I} + \Gamma + \Gamma^2 + \cdots = \sum_{m=0}^{\infty} \Gamma^m. \tag{7}$$

In order to relate the matrix P to the microscopic quantities, r and C, we use the fact that the identities

$$P_{KL} = \frac{1}{N}\sum_{k\in K, l\in L} B_{kl} = \sum_{k\in K} B_{kl'} \tag{8}$$

hold for any neurons l' in population L. These identities can be proven by considering each term in the infinite sum defining the matrix P, and noting that

$$[\Gamma^m]_{KL} = \frac{1}{N}\sum_{k\in K, l\in L} [G^m]_{kl}. \tag{9}$$

As an illustration of this identity, we consider the case $m = 2$; we have

$$\sum_{k\in K, l\in L} [G^2]_{kl} = \sum_{l\in L, k\in K, i\in I, I} G_{ki} G_{il} = \sum_{l\in L, i\in I, I} \Gamma_{KI} G_{il} = N \sum_{I} \Gamma_{KI}\Gamma_{IL} = [\Gamma^2]_{KL} N. \tag{10}$$

To express the firing rate of population I, R_I, we then use Eq. (8) to show that

$$R_I \equiv \sum_{i\in I} r_i = \sum_{i\in I}\sum_{K}\sum_{k\in K} B_{ik} r_{\text{ext},k} = \sum_{K}\sum_{i\in I, k\in K} B_{ik} r_{\text{ext},k} = \sum_{K} P_{IK} N R_{\text{ext},K}, \tag{11}$$

since $r_{\text{ext},k} = R_{\text{ext},K}$ for any $k \in K$. In vector form, this can be rewritten as the identity

$$R = P N R_{\text{ext}} = (\mathbb{I} - \Gamma)^{-1} N R_{\text{ext}}, \tag{12}$$

which matches Eq. (4). We can apply a similar manipulation to derive the covariances of the

population responses, Σ, as

$$\begin{aligned}\Sigma_{KL} &\equiv \sum_{k\in K, l\in L} C_{kl} \\ &= \sum_I \sum_{k\in K, l\in L, i\in I} B_{ki} B_{li} r_i \\ &= \sum_{l\in L} \sum_I \sum_{i\in I} P_{KI} B_{li} r_i \\ &= \sum_{I, i\in I} P_{KI} P_{RI} r_i \\ &= \sum_I P_{KI} P_{RI} R_I, \end{aligned} \tag{13}$$

which matches Eq. (5). (In Eq. (5), $D[R]$ is the diagonal matrix with $D[R]_{IJ} = \delta_{IJ} R_I$.)

3 Macroscopic signal and noise correlations as functions of connection strengths

We now evaluate signal and noise correlations in the population activity, as functions of the network couplings. The strength of the cross-coupling between populations, Γ_c, determines the population rates and covariances via the 'effective cross-coupling', P_c, in the population transfer matrix:

$$P = (\mathbb{I} - \Gamma)^{-1} = \frac{1}{(1-\Gamma_s)^2 - \Gamma_c^2} \begin{pmatrix} 1-\Gamma_s & \Gamma_c \\ \Gamma_c & 1-\Gamma_s \end{pmatrix} \equiv \begin{pmatrix} P_s & P_c \\ P_c & P_s \end{pmatrix}. \tag{14}$$

The network is unstable if the eigenvalues of Γ are larger than unity; in other words, stability requires $\Gamma_s + \Gamma_c < 1$, or $P_c < P_s$.

In a heterogeneous model with random coupling strengths, the signal and noise covariances were traced back to the mean and variance of the elements of the effective couple matrix. Similarly, here in a two-population model, the population behavior is ruled by the mean and variance of the elements of the matrix P, or, stated more simply, the two quantities $P_s + P_c$ and $P_s - P_c$. To make this statement more precise, we consider an ensemble of two-component input vectors, $R_{\text{ext}}(s) = \left(r_{\text{ext}}(s), r_{\text{ext}'}(s)\right)^T$, with the random external inputs, r_{ext} and $r_{\text{ext}'}$, as elements and chosen from a normal distribution with with mean $\mu_{r_{\text{ext}}}$ and variance $\sigma^2_{r_{\text{ext}}}$ independently across a set of stimuli, s. The average population response, $R = PR_{\text{ext}}$, then yields signal covariances, $\Sigma^S_{ij} = cov(R_i, R_j)$, across the stimulus ensemble, given by

$$\Sigma^S = \sigma^2_{r_{\text{ext}}} \begin{pmatrix} P_s^2 + P_c^2 & 2P_sP_c \\ 2P_sP_c & P_s^2 + P_c^2 \end{pmatrix}. \tag{15}$$

And the average signal correlation for the population activity is calculated as

$$\begin{aligned}\langle c_S\rangle &= \frac{1}{4}\sum_{ij}\frac{\Sigma^S_{ij}}{\sqrt{\Sigma^S_{ii}\Sigma^S_{jj}}}\\ &= 1+\frac{2P_sP_c}{P_s^2+P_c^2}\\ &= \frac{(P_s+P_c)^2}{(P_s+P_c)^2+(P_s-P_c)^2}\\ &= \frac{\langle P\rangle^2}{\langle P\rangle^2+\mathrm{var}(P)}\\ &= \frac{1}{1+\mathrm{var}(P)/\langle P\rangle^2},\end{aligned} \tag{16}$$

where we have used the notation

$$\langle P\rangle \equiv \frac{P_s+P_c}{2} \tag{17}$$

and

$$\mathrm{var}(P) \equiv \frac{1}{2}\left[\left(P_s-\frac{P_s+P_c}{2}\right)^2+\left(P_c-\frac{P_s+P_c}{2}\right)^2\right]=\left(\frac{P_s-P_c}{2}\right)^2. \tag{18}$$

From Eq. (16), the normalized variance of the elements of P determines the strength of signal correlations. It is small if the network is strongly connected, that is, if Γ_c is large.

A similar relation holds for the average noise correlations, if we average first over stimuli, then over neurons: the average of the noise covariance matrix over stimuli is $\langle\Sigma(s)\rangle_s = \langle PR(s)P^T\rangle = P\langle R\rangle P^T$. Because the external input to both populations is identical, $\langle r_{\mathrm{ext}}\rangle = \langle r'_{\mathrm{ext}}\rangle$, the average rates of both populations are identical, and the average over population pairs can be evaluated just as in the case of the signal correlations. From

$$\langle\Sigma(s)\rangle_s = \langle r_{\mathrm{ext}}\rangle\begin{pmatrix}P_s^2+P_c^2 & 2P_sP_c\\ 2P_sP_c & P_s^2+P_c^2\end{pmatrix}, \tag{19}$$

it follows that

$$\langle c_N\rangle = \frac{1}{4}\sum_{ij}\frac{\langle\Sigma\rangle_{ij}}{\sqrt{\langle\Sigma\rangle_{ii}\langle\Sigma\rangle_{jj}}}=\frac{1}{1+\mathrm{var}(P)/\langle P\rangle^2}. \tag{20}$$

4 Condition on circuit parameters for the emergence of beneficial correlations

In the context of the two-population model, we can show that, depending on the cross-coupling, noise correlations can be beneficial for stimulus discrimination. This is meant in a comparison to the case of independent neurons, obtained by shuffling trials. As an illustration, consider a situation in which the population responses to two stimuli, s_1 and s_2, are $R(s_1) = (R_0+\Delta R, R_0)$ and $R(s_2) = (R_0, R_0+\Delta R)$, respectively. For the sake of simplicity, we assume that the population covariance matrix is stimulus-independent,

$$\Sigma(s_1)=\Sigma(s_2)=\begin{pmatrix}\Sigma_{EE} & \Sigma_{E'E}\\ \Sigma_{E'E} & \Sigma_{E'E'}\end{pmatrix}, \tag{21}$$

and $\Sigma_{EE}=\Sigma_{E'E'}$ Here, the most discriminant direction (see Eq. (M21), w, here is an eigenvector of Σ, $(1,-1)$, and the variance along this direction corresponds to the smaller of the two eigenvalues of the matrix Σ, namely

$$\sigma_w^2 = \Sigma_{EE}-\Sigma_{E'E} = \sum_{i\in E}C_{ii}+\sum_{i\neq j\in E}C_{ij}-\sum_{i\in E,k\in E'}C_{ik}. \tag{22}$$

If this quantity is smaller than the population variance in a two-population model with independent neurons, namely $\sigma^2_{\text{indep}} = \sum_{i\in E} C_{ii}$, then noise correlation are beneficial to stimulus discrimination. See Fig. 1 B-E for an illustration from a numerical calculation. From Eq. (22), this condition is equivalent to requiring that the summed covariances across the two populations, $C_{cross} = \sum_{i\in E,k\in E'} C_{ik}$, be larger than the summed covariances within a population, $C_{within} = \sum_{i\neq j\in E} C_{ij}$.

We can now translate this condition on the values of covariances to a condition on the values of circuit couplings in our dynamical model. For the sake of simplicity, we consider the situation in which $R_{\text{ext}} = R_{\text{ext}'} \equiv R$; then

$$\Sigma = \begin{pmatrix} \Sigma_{EE} & \Sigma_{E'E} \\ \Sigma_{E'E} & \Sigma_{E'E'} \end{pmatrix} = R \begin{pmatrix} P_s^2 + P_c^2 & 2P_sP_c \\ 2P_sP_c & P_s^2 + P_c^2 \end{pmatrix}. \tag{23}$$

The average pairwise population cross-covariance is calculated as $C_{cross} = \Sigma_{E'E}/N^2$. Approximately, the average neuron variance differs from the average within-covariance only by the contribution of the rate to the variance: $\langle C_{ii}\rangle \approx R/N + C_{within}$(this can be seen from a series expansion of the equation for the covariances, $C = BD[R]B^T$). From this we get for the population variance $\Sigma_{EE} = N\langle C_{ii}\rangle + N(N-1)C_{within} \approx R + N^2 C_{within}$. With Eq. (23), the condition on covariances, $C_{cross} > C_{within}$, translates into the following condition on circuit parameters:

$$\frac{2P_sP_cR}{N^2} > \frac{R(P_s^2 + P_c^2) - R}{N^2}, \tag{24}$$

or

$$2P_sP_c > (P_s^2 + P_c^2) - 1, \tag{25}$$

or

$$(P_s - P_c)^2 < 1. \tag{26}$$

Since $P_s > P_c$, we obtain the simple inequality

$$P_s - P_c < 1. \tag{27}$$

Using Eq. (14) for the elements of the matrix P, we obtain the condition

$$\frac{1 - \Gamma_s - \Gamma_c}{(1-\Gamma_s)^2 - \Gamma_c^2} < 1 \Leftrightarrow \Gamma_s(1-\Gamma_s) > \Gamma_c(1-\Gamma_c).,\tag{28}$$

or

$$f(\Gamma_s) > f(\Gamma_c), \tag{29}$$

where the function $f(x) = x(1-x)$ is a parabola that goes through the points $(0,0)$ and $(0,1)$, and has its minimum at $(1/2, -1/4)$. Since $1 - \Gamma_s > \Gamma_c$ (see Eq. (14)), the condition in Eq. (29) can be fulfilled if $\Gamma_s < 1/2$ and within-coupling is smaller than cross-coupling, $\Gamma_s < \Gamma_c$.

5 Continuous stimuli changing in arbitrary directions

Above, we used the signal-to-noise ratio, S, to measure how well discrete pairs of stimuli in a given ensemble can be discriminated. In a model network, we have access to all possible input dimensions, and we can use an alternative measure that combines these possible stimulus dimensions. If a high-dimensional stimulus varies continuously, the information in the response distribution about the stimulus can be measured by the Fisher information matrix [3]. An approximation, the 'linear Fisher information', is obtained by assuming Gaussian noise and by neglecting the information pertaining to the stimulus dependence of the covariance matrix; the elements of the linear Fisher Information matrix are defined as

$$I_{mn} = \partial_m R^T \Sigma^{-1} \partial_n R, \tag{30}$$

for the population covariance matrix, Σ, and population response, R. Here, $\partial_m R$ is the derivative of the response vector with respect to stimulus coordinate m. Our stimulus dimensions are the coordinates of the input vector, R_{ext}, and the mean output vector, in a network model, is $R = PNR_{\text{ext}}$. It is easy to see that $\partial_m R$ is given by the mth column of P, and, hence,

$$I = P^T \Sigma^{-1} P. \tag{31}$$

As the covariance matrix in a recurrent network is given by $\Sigma = PD[R]P^T$, the corresponding linear Fisher Information matrix reads

$$I = D[R]^{-1}. \tag{32}$$

According to Eq. 32, the linear Fisher information scales as the inverse of the strength of connections in the network. This is a special case of the more general result considered in [4]. This obtains because recurrent connections enhance noise, as compared to the firing rates. Generally, if the input depends linearly on a one-dimensional stimulus s, $R_{\text{ext}}(s) = R_{\text{ext},0} + s\Delta R_{\text{ext}}$, the information about changes in this direction is $\Delta R_{\text{ext}}^T I \Delta R_{\text{ext}}$. As a measure of the information about stimulus changes in all possible directions, we use the trace of I, $\text{Tr}(I) = \sum_i I_{ii}$.

6 Impact of circuit properties on stimulus coding: intuition from the two-population model

We now exploit the various observations summarized above to investigate in greater detail the effect of the circuit parameters on the accuracy of population coding, in the context of our simple, two-population model. We start by examining how the cross-coupling between the two populations influences the statistics of the responses to two stimuli. Upon increasing the cross-coupling, Γ_c, the response distributions evoked by the two stimuli change in a specific manner (Fig. 1): the average population responses are enhanced, but also more similar, i.e., the difference in the population responses to the two stimuli, $|R(s_1) - R(s_2)|$, is suppressed. At the same time, the noise correlation between the two population responses is enhanced, so that the two response distributions become 'more elongated', Fig. 1 B.

To examine the combined impact of these changes on stimulus coding, we compare this situation to that of a population of independent neurons with matched average responses and variances; the latter can be obtained simply by shuffling the data from the correlated population among trials. As we showed in the sections above, a condition for correlations that are beneficial to stimulus coding, from this angle, is that correlations between neurons in different populations be stronger than pairwise correlations within the same population. This condition is realized for strong coupling between the two populations (Fig. 1 C,D). Considering the dependence of the signal-to-noise ratio, S (the difference in means divided by the variance, see Eq. (M22) in the main text), on the cross-coupling, we observe that increasing the cross-coupling between the two populations decreases the signal-to-noise ratio, in both the 'native' and the shuffled cases (Fig. 1 E); but this suppression is stronger in the shuffled case. The suppression can be traced back to the noise produced in spike generation: under amplification through a matrix, P, firing rates, $R = PNR_{\text{ext}}$, scale linearly with P, while the covariances, $\Sigma = PD(R)P^T$, scale with a higher power of P. In our model, this cubic dependence on P comes from the combination of rate fluctuations (resulting from recurrent inputs from Poisson neurons and yielding a quadratic contribution) and the Poisson spike generation. Because of the cubic dependence of the variances, as compared to the linear dependence of the rates, the signal-to-noise ratio decreases as a function of the strength of the recurrent connections, P.

Independent of this effect, larger cross-coupling reshapes both external and internally generated noise to relegate it along a direction that is irrelevant for discrimination. This

mechanism would compensate for the decrease in the difference of the average responses, if no internal noise were generated. This gain is reduced when correlations are shuffled, such that more information is lost in this case.

Up to here, we considered the special case of two stimuli. For a larger set of stimuli, noise correlations may be harmful for discriminating some of the stimulus pairs, while beneficial for discriminating others (as compared to the case of independent neurons). In Fig. 1 F, an example is sketched for each of the two cases: a pair of stimuli where both populations receive an identical input in the case of each stimulus (connected by a dashed line), and a pair of stimuli where in each case one population receives stronger input (connected by a dotted line). For an ensemble of stimuli, the overall effect of recurrent amplification depends on the distribution of favorable and unfavorable pairs. In order to quantify the effect of recurrent amplification more generally, rather than averaging across a number of possible stimulus pairs, we consider a measure that takes into account only local changes in the stimulus, but in all possible directions. In the context of the scenario visualized in Fig. 1 F, we consider a stimulus which can result in an average response at the position where the dashed and the dotted lines cross. To measure the combined effects on information for the case in which a change in stimulus results in a change of the response along any line in the plane, we use the trace of the linear Fisher information matrix (see the previous section for details). In Fig. 1 G, we show how the trace of the linear Fisher information matrix decreases with the strength of recurrent amplification, which we measure by the average over the elements of the matrix P.

7 Impact of circuit properties on stimulus coding in an extended model with excitatory and inhibitory neurons

The simple two-population model considered above comes with a limited set of parameters. It is instructive to examine a slightly extended model, with four coupled neural sub-populations: two excitatory sub-populations (E_1 and E_2) and two inhibitory sub-populations (I_1 and I_2). In this model, we consider connections between neurons within each excitatory sub-population (resulting in population coupling $E_1 \longleftrightarrow E_1$ and $E_2 \longleftrightarrow E_2$ equal in magnitude), cross-connections between the neurons from the two excitatory sub-populations ($E_1 \longleftrightarrow E_2$), and connections between pairs of neurons from the excitatory and inhibitory sub-populations ($E_1 \longleftrightarrow I_1$ and $E_2 \longleftrightarrow I_2$). (In our simulations, each of these sub-populations contains 100 neurons.)

As in the two-population model discussed above, this model can be described by effective parameters which combine the size of sub-populations, synaptic strengths, and the probabilities of forming synapses; two important parameters are the self-coupling within E_1 and E_2, Γ_S, and the cross-coupling between E_1 and E_2, Γ_C. By appropriately choosing the other parameters in the model, we can keep the firing rates in E_1 and E_2, as well as the single-neuron variance averaged over E_1, unchanged as we vary Γ_S and Γ_C. (It would be possible to keep also the variance in E_2 invariant, by extending the model further to include an additional parameter, such as inhibitory self-connections. We do not follow this route, here, for the sake of conciseness.)

In spite of its simplicity, the model with both excitatory and inhibitory neurons exhibits an intriguing behavior in term of stimulus coding. We can examine the behavior of this model using a framework similar to the one described above. Here, we present the corresponding results, in terms of the signal-to-noise ratio for the discrimination between two stimuli (defined as before). The signal-to-noise ratio, S, varies as a function of both Γ_S and Γ_C; it increases for smaller Γ_S and larger Γ_C (2 A). Furthermore, fixing the population-averaged variance to a set of values (2 A, top) defines a set of curves in the (Γ_S, Γ_C)-plane (2 A,

bottom). These demonstrate that, even in the presence of fixed variance, circuit parameters can be varied in such a way as to modulate the signal-to-noise ratio appreciably. Interestingly, a higher signal-to-noise ratio does not result from a reduced noise entropy: indeed, the noise entropy of the correlated population in fact grows for larger signal-to-noise ratios, as compared to the noise entropy in a matched independent population (2 B)

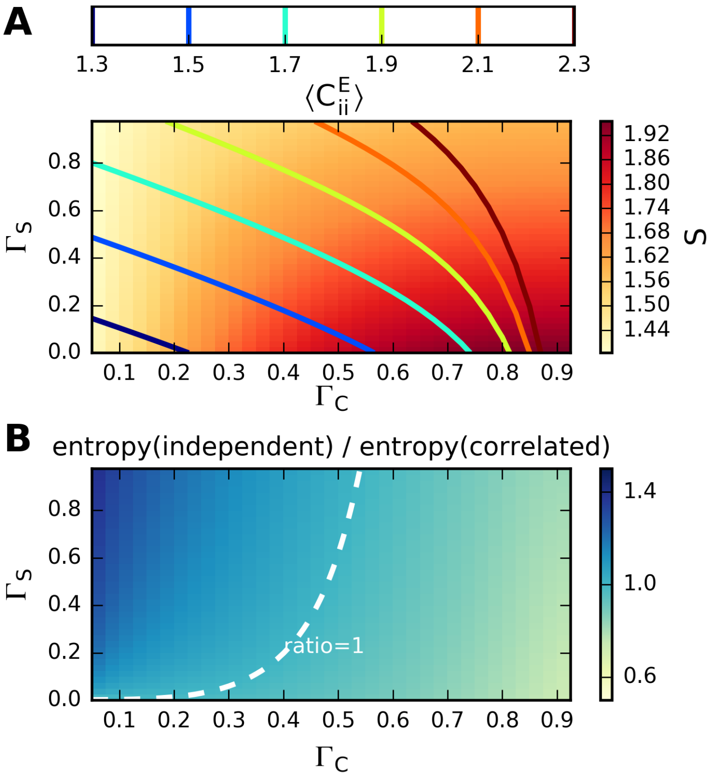

Fig 2. Effects of circuit parameters on stimulus coding in a simple recurrent network. A: Signal-to-noise ratio as a function of the self-coupling, Γ_S, and the cross-coupling, Γ_C, in two excitatory sub-populations (bottom). For a set of fixed population-averaged variances (top), the allowed values of the parameters Γ_S and Γ_C describe curves along which the signal-to-noise can vary appreciably (bottom). B: Ratio of the noise entropy in a model of independent neurons with matched firing rates and population-averaged variance and and the noise entropy in the recurrent model, as a function of the parameters Γ_S and Γ_C.

References

1. Pernice V, Staude B, Cardanobile S, Rotter S. How Structure Determines Correlations in Neuronal Networks. PLoS Comput Biol. 2011;7(5):e1002059. doi:10.1371/journal.pcbi.1002059.

2. Trousdale J, Hu Y, Shea-Brown E, Josić K. Impact of network structure and cellular response on spike time correlations. PLoS Comput Biol. 2012;8(3):e1002408. doi:10.1371/journal.pcbi.1002408.

3. Abbott LF, Dayan P. The effect of correlated variability on the accuracy of a population code. Neural Comput. 1999;11(1):91–101.

4. Beck J, Bejjanki VR, Pouget A. Insights from a simple expression for linear fisher information in a recurrently connected population of spiking neurons. Neural Comput. 2011;23(6):1484–1502. doi:10.1162/NECO_a_00125.
