# Supplementary figures and images for: Interpretation of correlated neural variability from models of feed-forward and recurrent circuits
Source: PLoS Comput Biol. 2018 Feb 6;14(2):e1005979. doi: 10.1371/journal.pcbi.1005979 (PMC5833435; doi:10.1371/journal.pcbi.1005979)

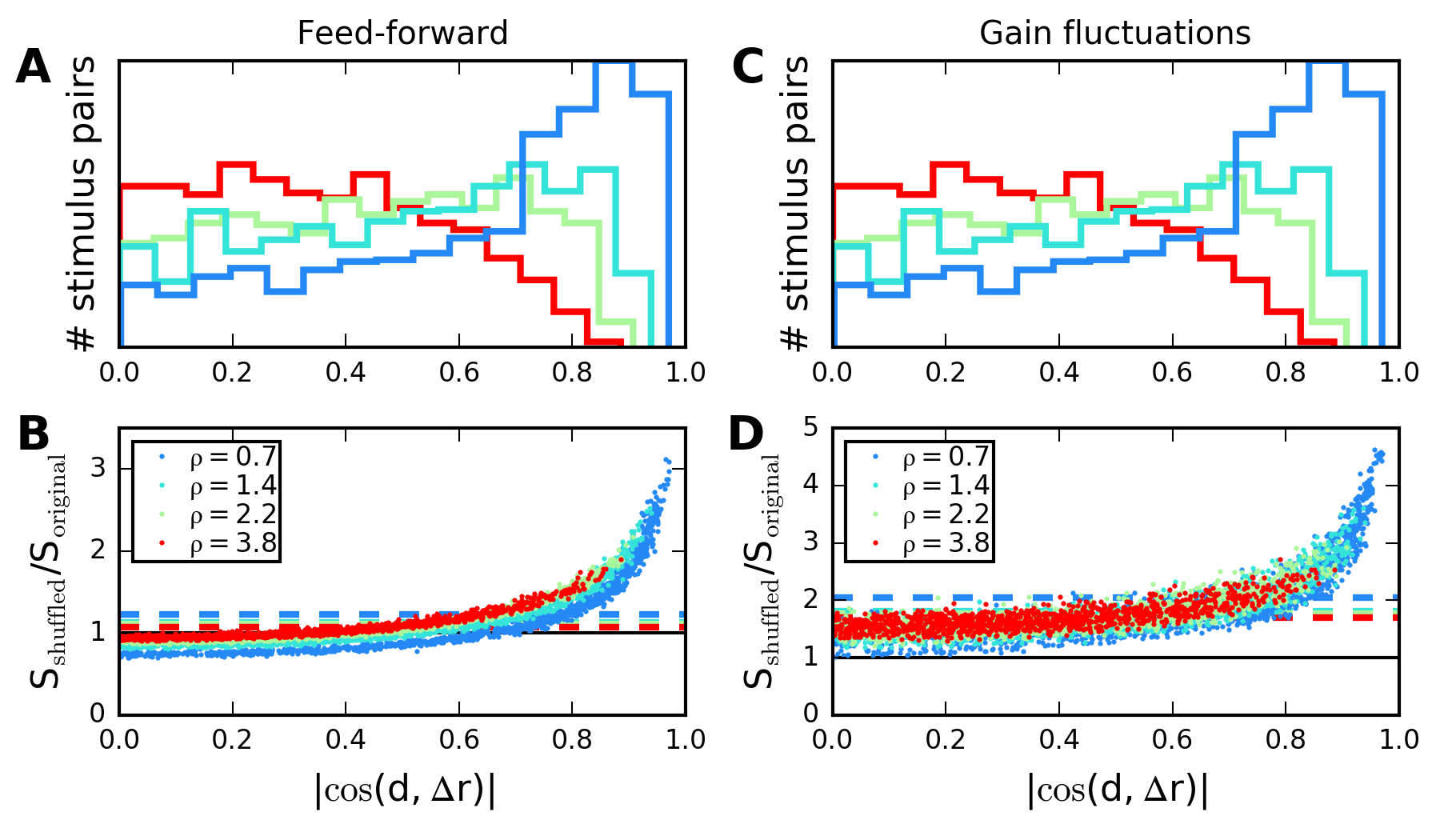

Supplement: S1 Fig — (Compare to Fig 4). Parameters for the feed-forward network with shared input are as in Fig 2. As average responses are arbitrary in the gain fluctuation model, identical average responses as in the shared input model were assumed, and the variance of gain fluctuations set to Vext = 0.53. A, C: Distributions of cosines of angle between the diagonal and the difference of average responses for stimulus pairs, in feed-forward networks with shared input (A) and common gain fluctuation (C), for different values of the parameter ρ. B, D: Discrimination ratios in the cases of correlations generated by shared input (B) and common gain fluctuation (D), for the same set of values of the parameter ρ. (TIF) [file pcbi.1005979.s003.tif]
